# Characterizing corn-straw-degrading actinomycetes and evaluating application efficiency in straw-returning experiments

**DOI:** 10.3389/fmicb.2022.1003157

**Published:** 2022-12-05

**Authors:** Xiujie Gong, Yang Yu, Yubo Hao, Qiuju Wang, Juntao Ma, Yubo Jiang, Guoyi Lv, Liang Li, Chunrong Qian

**Affiliations:** ^1^Institute of Farming and Cultivation, Heilongjiang Academy of Agricultural Sciences, Harbin, China; ^2^Heilongjiang Academy of Black Soil Conservation and Utilization, Harbin, China; ^3^Institute of Biotechnology, Heilongjiang Academy of Agricultural Sciences, Harbin, China

**Keywords:** corn straw decomposing, microbial consortium G123, genome analysis, straw returning, microbial diversity

## Abstract

Corn straw is an abundant lignocellulose resource and by-product of agricultural production. With the continuous increase in agricultural development, the output of corn straw is also increasing significantly. However, the inappropriate disposal of straw results in wasting of resources, and also causes a serious ecological crisis. Screening microorganisms with the capacity to degrade straw and understanding their mechanism of action is an efficient approach to solve such problems. For this purpose, our research group isolated three actinomycete strains with efficient lignocellulose degradation ability from soil in the cold region of China: *Streptomyces* sp. G1^T^, *Streptomyces* sp. G2^T^ and *Streptomyces* sp. G3^T^. Their microbial properties and taxonomic status were assessed to improve our understanding of these strains. The three strains showed typical characteristics of the genus *Streptomyces*, and likely represent three different species. Genome functional annotation indicated that most of their genes were related to functions like carbohydrate transport and metabolism. In addition, a similar phenomenon also appeared in the COG and CAZyme analyses, with a large number of genes encoding carbohydrate-related hydrolases, such as cellulase, glycosidase and endoglucanase, which could effectively destroy the structure of lignocellulose in corn straw. This unambiguously demonstrated the potential of the three microorganisms to hydrolyze macromolecular polysaccharides at the molecular level. In addition, in the straw-returning test, the decomposing consortium composed of the three *Streptomyces* isolates (G123) effectively destroyed the recalcitrant bonds between the various components of straw, and significantly reduced the content of active components in corn straw. Furthermore, microbial diversity analysis indicated that the relative abundance of *Proteobacteria*, reportedly associated with soil antibiotic resistance and antibiotic degradation, was significantly improved with straw returning at both tested time points. The microbial diversity of each treatment was also dramatically changed by supplementing with G123. Taken together, G123 has important biological potential and should be further studied, which will provide new insights and strategies for appropriate treatment of corn straw.

## Introduction

Corn (*Zea mays*) is a vitally important feed and food resource that is cultivated worldwide. As the world’s largest producer of straw, China now faces momentous challenges in dealing with agricultural residues. A mathematical programming model, used to estimate the economic value of biomass supply from crop residues, revealed that China can produce about 174.4–248.6 million metric tons of agricultural residues annually, with corn straw accounting for 28% of this (Chen, 2016), followed by rice and wheat straw. Due to its slow degradation rate, agricultural straw is increasingly disposed of inappropriately or burned directly, which not only results in waste of resources but also has a negative effect on the environment (Hong et al., 2016; Liu et al., 2020; Wang et al., 2021). Open burning of straw has been identified as one of the largest sources of anthropogenic pollution, with a significant effect on the quality of local air and contributing to generation of regional haze. The incremental release of PM2.5 (aerodynamic diameter of particulate matter ≤2.5 μm) is the principal reason for the formation of fog and haze, and the air pollution attribute to PM_2.5_ is the greatest threat to human health in China (Qu et al., 2012). The rational utilization of corn straw resources and the reduction of environmental pollution are urgent tasks, which not only relate to the sustainable development of agriculture but also to environmental safety. Crop straw contains abundant organic matter, including cellulose, hemicellulose, lignin, protein and ash. Straw returning technology is a strategic choice for protecting the environment and promoting sustainable agricultural development. It has a positive impact on protecting the soil ecological environment, improving soil structure, preventing soil erosion, enhancing microbial biodiversity, and diversifying the nutrient supply including of organic matter, nitrogen, phosphorus, calcium, magnesium, and potassium in farmland ecosystems (Benbi and Senapati, 2010; Song et al., 2020). However, the main disadvantage of corn straw returning is incomplete degradation of straw because of insufficient treatment time and sluggish decomposition rate under natural conditions, which hinders root penetration and aggravates crop pest infestation (Li et al., 2018; Ren et al., 2019; Ma et al., 2020). Particularly in Heilongjiang Province in northeast China, owing to the long freezing time in winter, low accumulated temperature of soil and the dry climate, decomposition rate of returned straw is slower than in other regions. Therefore, accelerating the development and application of straw biodegradation technology, which depends on biodegradation by microorganisms such as actinomycetes, is extremely important for the direct return and utilization of straw and the reduction of environmental pollution caused by straw burning (Hatamoto et al., 2008).

*Actinobacteria* species, which are widespread in terrestrial ecosystems (Maciejewska et al., 2015; Li et al., 2016; Law et al., 2017), are the most agriculturally and biologically valuable prokaryotes. *Streptomyces* is the most representative and dominant genus of the phylum *Actinobacteria* (Kämpfer and Labeda, 2006). *Streptomyces* spp. have become a popular bioprospecting subject due to the extensive range of biological activities of the secondary metabolites, such as insecticides milbemycins (He et al., 2018), immunosuppressive rapamycin (Kim et al., 2014), antibacterial formicamycins (Qin et al., 2017) and antifungal piericidin (Ortega et al., 2019). These properties endows such microorganisms with the natural advantage of maintaining activity in various special habitats or presence of antagonistic species. In addition to antibiotics, *Streptomyces* strains are prolific producers of extracellular enzymes involved in lignocellulose biodegradation (Xu and Yang, 2010; Prakash et al., 2013; Saini and Aggarwal, 2018). The enzymes associated with the degradation of lignocellulose comprise endoxylanases, amylases, peroxidases, cellulases, hemicellulases and ligninases, which can effectively hydrolyze the cellulose, hemicellulose and lignin that are the main components of plant cell walls. Cellulases have received worldwide attention because of their enormous potential in various industrial applications through transforming cellulosic biomass into available sugar which can be used by microorganisms to generate other value-added products, such as bioethanol and biomethane (Sharma et al., 2016; Mihajlovski et al., 2021). Multiple types of hemicellulases have been isolated from mesophilic and thermophilic microorganisms, and their predominant applications are pulp bio-bleaching and deinking (Soni and Kango, 2013). Lignin, the second most abundant renewable carbon source on earth after cellulose, is found in all vascular plants. It can protect the structural polysaccharides in the cell walls and tissues from attack by phytopathogens, because its recalcitrant, complex chemical structure, and linkage heterogeneity (Abdel-Hamid et al., 2013). Peroxidase and laccases identified from *Streptomyces* spp. can efficiently depolymerize lignin to produce low molecular weight, water-soluble, acid-precipitable aromatic compounds and release single-ring aromatic phenols (Riyadi et al., 2020). In addition, although different microorganisms can generate lignocellulases, actinomycetes are the most advantageous producers because they not only produce abundant hydrolytic enzymes, but also have strong tolerance and adaptability to extremes such as acidic, alkaline, hyperhaline and hyperthermal environments (Dastager et al., 2008; Zhang et al., 2013; Hui et al., 2021). Therefore, bioprospecting for novel *Streptomyces* taxa is important for industrial and agricultural production.

In recent years, some strains with the ability to degrade corn straw have been screened. However, most of them have the problem of low enzyme activity with ignoring whether the strain has the ability to degrade the other two main components (hemicellulose and lignin) that significantly reduce the utilization rate of cellulose. Based on the above research foundation，a microbial consortium, with a positive effect on corn straw degradation (Gong et al., 2020), was screened by our research group. To provide a comprehensive understanding of these biodegradable strains, their morphological properties, chemotaxonomy and physiological and biochemical characteristics were investigated. A phylogenetic tree was constructed for use in determining the taxonomic status of the relevant species. Whole-genome sequencing was utilized to interpret the molecular mechanisms of straw degradation and evaluate the biological potential of decomposing strains. In addition, in this study, the impacts of native microorganisms in the soil, commercial decomposing agents and specific microbial flora on decomposition rate of straw components were compared for different treatment times. Corn straw specimens, following digestion by different treatment groups, were characterized by scanning electron microscopy (SEM), Fourier transform-infrared spectroscopy (FTIR) and X-ray diffraction (XRD). We also determined the effects of straw returning on soil bacterial communities by utilizing Illumina-based 16S rRNA amplicon sequencing.

## Materials and methods

### Strains and growth conditions

Existing research has clarified that humus is mainly formed by the action of microorganisms, so it is a significant source for isolation of cellulose- and lignin-degrading species (Wang et al., 2019; Kong et al., 2022). Considering this, we screened humus samples collected from multiple cold regions in Heilongjiang Province: Wudalianchi City, 48.17 °N, 126.35 °E; Xunke County, 47.58 °N, 129.17 °E; and Bei’an City, 47.35 °N, 127.51 °E. *Streptomyces* sp. G1^T^, *Streptomyces* sp. G2^T^ and *Streptomyces* sp. G3^T^ (hereafter G1, G2 and G3, respectively), which have promising activity, were obtained and deposited in the China General Microbiological Culture Collection Center (patent preservation numbers CGMCC No. 19310, 23,358 and 23,359, respectively). All tested organisms were incubated at 28°C statically or shaken at 180 rpm in ISP2 liquid medium: 0.4% glucose, 1% malt extract, 0.4% yeast extract and 2% agar at pH 7.0–7.4. The effective number of living bacteria exceeded 1 × 10^9^ cfu/ml. For spore preparation and morphological comparison, G1, G2 and G3 were cultivated at 28°C on ISP3 medium (Shirling and Gottlieb, 1966) containing 20 g of oat powder, 0.2 g of MgSO_4_·7H_2_O, 0.2 g of KNO_3_, 0.5 g of K_2_HPO_4_, 15 g of agar and 1,000 ml of water. For decomposition of corn straw, “Wobao” brand degradation bacteria agent, purchased from Henan Wobao Biotechnology Co., Ltd., was employed as the control group. The number of effective living bacteria exceeded 1 × 10^9^ cfu/g. The amount of decomposing agent used was 0.25 g per 100 g of corn straw.

### Strain characterization

Morphological properties were observed by light microscopy (Nikon ECLIPSE E200) and electron microscopy (JSM-6700F) after the isolates were statically incubated on ISP3 agar medium at 28°C for 28 days. Samples for SEM were prepared as follows. Specimens were prepared by cutting a square (0.5 cm × 0.5 cm) from the ISP3 agar plate, fixed in 2.5% glutaraldehyde solution and dehydrated with 50, 75, 80, 90, 95 and 100% ethanol, respectively. Subsequently, all samples were replaced by passing tert-butanol, and then critical-point drying using a lyophilizer. The dried samples were placed onto a stub bearing adhesive, sputter coated with a gold layer and viewed using SEM. Spore motility was assessed by light microscopy (Nikon ECLIPSE E200) observation of cells suspended in phosphate buffer (pH 7.0, 1 mM). In addition, tolerance to various NaCl concentrations (0–10% [w/v] at intervals of 1% unit; determined at 28°C) and pH (4.0–12.0 at intervals of 1.0; determined at 28°C) of G1–G3 were cultured in ISP2 broth was tested for 2–3 weeks on a rotary shaker (Zhao et al., 2019). To investigate optimum growth conditions of the three tested strains, the temperature range for growth was 5–45°C with intervals of 5°C. The utilization of sole carbon and nitrogen sources was determined according to Kämpfer et al. (1991). Hydrolysis of cellulose, starch, aesculin, gelatin and Tween (20, 40 and 80), milk peptonization and coagulation, nitrate reduction and H_2_S production were examined as described previously (Gordon et al., 1974; Yokota et al., 1993). Production of catalase and urease was tested as described by Smibert and Krieg (1994).

Biomass and freeze-dried cells of G1–G3 for chemotaxonomic analysis were acquired and analyzed in triplicate after cultivating in ISP2 broth at 28°C for 7 days in shaken flasks (shaking at about 180 rpm). To determine cellular fatty acid compositions, biomass was collected by centrifugation at 10000 rpm for 10 min, thoroughly washed with sterile distilled water three times, and then discarded the supernatant for subsequent studies. Fatty acid methyl esters were investigated following the methodology of Gao et al. (2014) and analyzed by GC–MS using the procedure of Xiang et al. (2011). Cell mass for the whole-cell sugars analysis was hydrolyzed by hydrochloric acid, and the products detected according to the protocols developed by Lechevalier and Lechevalier (1980) and analyzed by thin-layer chromatography (TLC) according to Staneck and Roberts (1974). Phospholipids in cells were extracted using a mixture of chloroform, methanol and 0.3% aqueous NaCl, followed by shaking at 28°C for 2–3 h and pipetting the chloroform phase, which contained the target detection component (Minnikin et al., 1984). Separation was by two-dimensional TLC with the first direction developed in chloroform:methanol:water (65,25,4, v/v) and the second in chloroform:methanol:acetic-acid:water (80,18,12,5). Total lipid material was examined utilizing molybdatophosphoric acid, and specific functional groups were detected employing special spray reagents such as trione ninhydrin, molybdenum blue and alpha-naphthol reagent (Collins and Jones, 1980). Menaquinones were derived from lyophilized material and purified according to Collins (1985), and analysis was performed using an Agilent TC-C18 Column (250 × 4.6 mm i.d. 5 μm) at a detection wavelength of 270 nm using the high-performance liquid chromatography-UV method described by Wu et al. (1989).

### Phylogenetic analyses

Determination of phylogenetic neighbors and similarity analysis were accomplished exploiting the EzBioCloud server (Yoon et al., 2017). Multiple alignments of the 16S rRNA sequences of the three tested strains were carried out with representative sequences of closely related organisms in the genus *Streptomyces* obtained from the GenBank/EMBL/DDBJ databases using Clustal X 1.83 software. Phylogenetic trees were produced with neighbor-joining (Saitou and Nei, 1987) algorithms using Molecular Evolutionary Genetics Analysis (MEGA) software version 7.0. Bootstrap analysis was performed based on 1,000 resampling to ensure stability of the resultant tree topologies (Felsenstein, 1985).

### Genome sequencing, assembly, and function analysis

Genomic DNA of G1–G3 was extracted with the SDS protocols (Lee et al., 2003). Tested species were grown for 7 days in ISP2 broth at 28°C and 180 rpm, and collected by centrifugation. The obtained cells were lysed frozen in liquid nitrogen and grinded into powder. Subsequently, resuspension of cells in buffer and treated with SDS. The extracted DNA was purified using a phenol/chloroform mixture, and precipitated in isopropanol. The harvested DNA was detected by agarose gel electrophoresis and quantified by Qubit. An embedded 350-bp PCR amplified product connected with A-tailed and the paired-end adapters was used for the library construction. The whole-genome sequencing was performed on an Illumina HiSeq PE150 platform at Beijing Biomarker Technologies Co., Ltd., China. Following filtering out of the low-quality and low-complexity reads, all good quality paired-end reads were assembled utilizing SOAPdenovo^1^ to generate the scaffolds (Li et al., 2008, 2010). The genomic DNA G + C content was calculated from the draft genome sequences of G1–G3. To explore the biological potential of the three strains with straw degradation ability, we performed multi-aspect functional prediction on the sequencing results, including using Gene Ontology (GO), Kyoto Encyclopedia of Genes and Genomes (KEGG) and Clusters of Orthologous Genes (COG) databases and carbohydrate-active enzymes annotation (CAZyme) (Ashburner et al., 2000; Kanehisa et al., 2004; Galperin et al., 2015). Furthermore, mining of the biosynthetic gene clusters for potential or unexplored secondary metabolites was performed employing “antibiotics and secondary metabolite analysis shell” (antiSMASH) (Blin et al., 2017).

### Straw-returning test of microbial consortium G123

The highly efficient corn straw degradation actinomycetes G1–G3, isolated from humus in a cold region, comprised a microbial consortium labeled G123 (Gong et al., 2020). Field application tests were used to better research the impact of G123 on corn straw degradation. There were three treatments in field experiments for corn straw returning: group 1 consisted of only soil and corn straw, to which was added 0.25% Wobao straw-decomposition agent and 3% microbial consortium G123 (three strains of cornstalk decomposing strains with complementary activities were mixed in equal proportions) for groups 2 and 3, respectively. Groups 1–3 are abbreviated SC-F, SCW-F and SCG-F, respectively; the standard sample of straw was labeled C. Firstly, 667 g of sifted bulk soil was put into a nylon mesh bag (34 cm × 36 cm) with an 80-mesh ventilation holes. Then, 20 g of corn straw, cut into 1 cm × 1 cm squares after drying at 70°C, was added into the bags. The added amounts of commercial degrading bacteria and microbial flora were consistent with the laboratory test. Sterile water was supplemented using a pipette to regulate the moisture content of each specimen to 60%. Finally, the nylon mesh bag was sealed with nylon joints and buried 20 cm deep in the soil along with a thermometer and hygrometer to monitor soil samples. Corn straw was taken twice (after about 11 and 17 weeks of incubation): samples at the first sampling were SC-F-I, SCW-F-I, and SCG-F-I, and at the second sampling were SC-F-II, SCW-F-II, and SCG-F-II.

### Corn straw degradation rates

The straw samples after various treatments were washed by the ultrasonic approach to remove the adhering soil to its surface, dried and then analyzed at multiple aspects. The corn straw degradation rate (Dec R) was calculated using the following equation. The weight loss for each treatment sample was used. Measuring the difference in organic contents between the treatment groups before or after a period was a simple approach used to evaluate the organic decomposition in both incubation and field experiments (Shiga et al., 1985; Goto et al., 2004). In the field experiments, Dec R was determined by the classical weight loss method:

$$Dec\;R\;\left(\%\right)=\frac{\left(\mathrm{original corn straw mass}-\mathrm{residual corn straw mass}\right)}{\mathrm{original corn straw mass}\times 100}$$

where residual corn straw before (original) and after decomposition was washed and dried at 80°C.

### Determining structure and composition of corn straw

The contents of cellulose, hemicellulose, lignin and ash in corn straw specimens, which were decomposed by different treatment groups, were determined by the procedure described by Van Soest et al. (1991). There were three repetitions of every sample. The FTIR (Thermo Scientific Nicolet IS5, United States) was used to analyze the changes of structures and components of corn straw samples after degradation by different experimental groups. Corn straw (1 mg) was thoroughly ground with 400 mg of KBr and laminated into pellets. The scanning wavelength was from 4,000 to 400 cm^−1^. An X-ray diffractometer (D8 advance, Bruker, Germany) was employed to record the crystallinity of straw samples with angle range of 5–90° (2θ) by step scanning with a diffractometer. Nickel-filter Cu-K (λ = 0.15417 nm) was the source of radiation, with scanning voltage of 40 kV and scanning current 30 mA. The crystallinity index (CrI) of the RSF was calculated according to the Segal empirical method (Zafeiropoulos et al., 2002; Tserki et al., 2005). The SEM was used for morphological characterization of materials. The samples were observed under 5-kV acceleration voltage, and 1,000× images were obtained to accurately reflect the structure and interface changes of corn straw samples. The samples preparation approach was consistent with that of the fungal cells.

### Community analysis using Illumina

Total bacterial DNA was extracted from the soils that obtained from different treatment groups using the manufacturer’s protocol (TIANamp Soil DNA Kit-181,121). The harvested DNA was assessed for quality and concentration by Nanodrop 2000. Purified DNA extracts were then submitted to the Molecular Research MR DNA laboratory (www.mrdnalab.com, Shallowater, TX, USA) for Illumina MiSeq sequencing of the bacterial 16S rRNA genes using the universal primer pair: 338F, (5′-ACTCCTACGGGAGGCAGCA-3′); and 806R, 5′-GGACTACHVGGGTWTCTAAT-3′. High-throughput sequencing analysis of microbiota in various specimens was performed based on the Illumina HiSeq 2,500 platform (2 × 250 paired ends) at Biomarker Technologies Corporation (Beijing, China). According to the relationship between the overlap and paired-end reads, raw tags were generated by the splicing sequence employing FLASH (version 1.2.11) (Magoč and Salzberg, 2011). Subsequently, the stitched sequences were denoised, processed and chimeras removed using Trimmomatic (version 0.33) and UCHIME (version 8.1) to obtain high-quality label sequences (Edgar et al., 2011; Bolger et al., 2014). Operational taxonomic units (OTUs) defined by clustering at 3% divergence were determined using USEARCH (version 10.0) with a 0.005% (percentage of all sequenced sequences) threshold. The OTUs were then annotated using the RDP Classifier (version 2.2 with a confidence threshold of 0.8) based on the Silva classification database. Finally, alpha-diversity (Mothur, version 1.30) and beta-diversity (QIIME, version 1.8.0) analyses were performed to compare the differences in microbial diversity among treatments.

## Results

### Morphological and physiological characteristics

In accordance with the prototypical properties of the genus *Streptomyces* (Williams, 1989), the 28-day-old culture of strains G1–G3 formed well-developed and extensively branched substrate mycelium, supporting the differentiation of aerial hyphae into spores. Non-motile, rectangular and single spores (0.5–0.6 × 0.9–1.1, 0.4–0.5 × 1.2–1.5 and 0.6–0.7 × 0.8–0.9 μm for G1–G3, respectively) with a smooth surface on aerial mycelium were observed (Supplementary Figure S1). Strains G1 and G2 generated open-loop spore chains (Supplementary Figures S1A,B), whereas G3 produced a spiral chain of spores (Supplementary Figure S1C). All three strains were positive for hydrolysis of starch and cellulose and production of catalase, but negative for hydrolysis of Tween 80, H_2_S formation and nitrate reduction. Strain G3 could, however, be unambiguously differentiated from G1 and G2 using milk peptonization and coagulation, liquefaction of gelatin and hydrolysis of Tween 40 and aesculin. These corn-straw-decomposing organisms could utilize L-arabinose, lactose, maltose, D-glucose, D-mannitol and D-mannose as sole carbon sources, as well as utilize L-alanine, L-serine and L-asparagine as sole nitrogen sources. Strains G1 and G3 could grow at 0–4% NaCl, but G2 at only 0–2%. Among the three strains, G2 had better tolerance to acid culture conditions. Other physiological and biochemical characteristics are summarized in Table 1. Thus, isolates *Streptomyces* sp. G1–G3 were considered to represent three disparate *Streptomyces* species.

**Table 1:** Physiological and biochemical properties among species *Streptomyces* sp. G1–G3.

**Characteristic**	**1**	**2**	**3**
Temperature range for growth (°C)	15–40	15–40	15–40
Maximum NaCl tolerance (%, w/v)	4	2	4
pH range for growth	5–11	6–10	6–11
**Hydrolysis of:**			
Starch	+	+	+
Aesculin	−	−	+
Tween 20	+	−	−
Tween 40	−	−	+
Tween 80	−	−	−
Liquefaction of gelatin	+	+	−
Nitrate reduction	−	−	−
Milk peptonization and coagulation	−	−	+
Production of H_2_S	−	−	−
Decomposition of cellulose	+	+	+
Urease	−	+	+
Catalase	+	+	+
**Carbon sources utilization:**			
L-arabinose	+	+	+
D-fructose	+	−	−
*meso*-inositol	−	−	−
Lactose	+	+	+
Maltose	+	+	+
Raffinose	−	+	+
L-rhamnose	+	−	+
D-ribose	+	−	+
D-galactose	−	+	+
D-glucose	+	+	+
D-mannitol	+	+	+
D-mannose	+	+	+
D-sorbitol	−	−	−
D-sucrose	−	−	+
D-xylose	−	−	−
**Nitrogen sources utilization:**			
L-alanine	+	+	+
L-arginine	−	−	+
Creatine	−	+	−
L-glutamine	+	−	+
L-proline	−	+	−
L-serine	+	+	+
L-threonine	−	+	−
L-tyrosine	−	+	−
L-asparagine	+	+	+
L-aspartic acid	−	+	−
L-glutamic acid	−	+	+
Glycine	+	+	−
**Chemotaxonomic characteristics**			
Polar lipids	DPG, PE, PG, PI, PIM, unidentified phospholipids and aminolipids	DPG, PG, PE, PI, PIM, unidentified phospholipids and aminolipids	DPG, PG, PE, PME, PC, PI, PIM and unidentified phospholipids
Menaquinones	MK-9(H_6_); MK-9(H_8_)	MK-9(H_6_); MK-9(H_8_)	MK-9(H_4_); MK-9(H_6_); MK-9(H_8_)
Whole-cell hydrolysates sugar	glucose and ribose	glucose and ribose	glucose and ribose

Strains: 1, *Streptomyces* sp. G1^T^; 2, *Streptomyces* sp. G2^T^, 3, *Streptomyces* sp. G3^T^. All data are from this study. +, Positive; −, negative.

### Chemotaxonomic analyses

The fatty acids profiles of G1–G3 are presented in Table 2. The major cellular fatty acids (percentage greater than 10%) in G1 and G2 were anteiso-C_15:0_ (25.83 and 25.12%, respectively), iso-C_16:0_ (18.28 and 15.90%) and C_16:0_ (14.14 and 14.34%); however, in G3 the major cellular fatty acids were iso-C_16:0_ (35.86%) and anteiso-C_15:0_ (10.64%). The fatty acid profile of the strains is in agreement with reported *Streptomyces* species; for instance *S. colonosanans* MUSC 93J^T^, *S. tardus* P38-E01^T^, *S. spinosus* SBTS01^T^ and *S. shenzhenensis* subsp. *oryzicola* W18L9^T^, which contain anteiso-C_15:0_ (21.3–26.6%) and iso-C_16:0_ (15.1–30.0%) as major fatty acids (Law et al., 2017; Králová et al., 2021; Kaewkla et al., 2022). The whole-cell sugars of G1–G3 were glucose and ribose (Supplementary Figures S2A–C). In G1 and G2, the predominant respiratory quinones were MK-9(H_6_) and MK-9(H_8_), whereas, G3 was distinguishable from the other two strains by lower amounts of MK-9(H_4_) (Supplementary Figures S2D–F). This finding is consistent with a report about multiple taxonomic identification of *S. benahoarensis* MZ03-37^T^, which was screened from a special habitat in a lava tube of La Palma (Gonzalez-Pimentel et al., 2022). The predominant polar lipids of G1 and G2 were diphosphatidylglycerol, phosphatidylethanolamine, phosphatidylglycerol, phosphatidylinositol, phosphatidyl inositol mannoside and other unidentified lipids, which were present in slightly different proportions in the two strains (Supplementary Figures S3A,B). However, the major polar lipids of G3 were diphosphatidylglycerol, phosphatidylglycerol, phosphatidylethanolamine, phosphatidylmethylethanolamine, phosphatidylcholine, phosphatidylinositol, phosphatidyl inositol mannoside and unidentified phospholipids (Supplementary Figure S3C).

**Table 2:** Cellular fatty acid composition (% of total) of cornstalk-decomposition strains *Streptomyces* sp. G1–G3.

Fatty acids	1	2	3
Saturated			
C_12:0_	–	–	2.55
C_14:0_	1.68	2.17	–
C_15:0_	1.06	1.33	0.84
C_16:0_	14.14	14.34	5.95
C_18:0_	1.01	–	–
Saturated branched			
iso-C_14:0_	5.07	4.57	9.68
iso-C_15:0_	6.89	8.27	7.59
iso-C_16:0_	18.28	15.90	35.86
iso-C_17:0_	2.20	2.61	2.74
iso-C_18:0_	0.72	0.97	3.6
anteiso-C_15:0_	25.83	25.12	10.64
anteiso-C_17:0_	7.19	6.55	4.80
C_17:0_ cyclo	6.07	8.82	2.72
Unsaturated straight			
iso-C_16:1_ H	3.43	3.54	5.36
anteiso-C_17:1_ ω9c	1.71	1.80	4.45
Summed Feature 3	3.21	2.28	1.71
Summed Feature 9	1.51	1.73	1.51

All the tested isolates were grown in ISP 2 broth in shake flasks at 28°C for 7 days. 1, *Streptomyces* sp. G1^T^; 2, *Streptomyces* sp. G2^T^; 3, *Streptomyces* sp. G3^T^. All data are obtained from this study. –, not detected or composition is less than 0.5% of the total fatty acids. Summed feature 3 contains C_16:1_ ω7c and/or C_16:1_ ω6c; summed feature 9 contains iso-C_17:1_ ω9c and/or 10-methyl C_16:1_.

### Phylogenic analysis of cornstalk decomposition species

**Figure 1:**
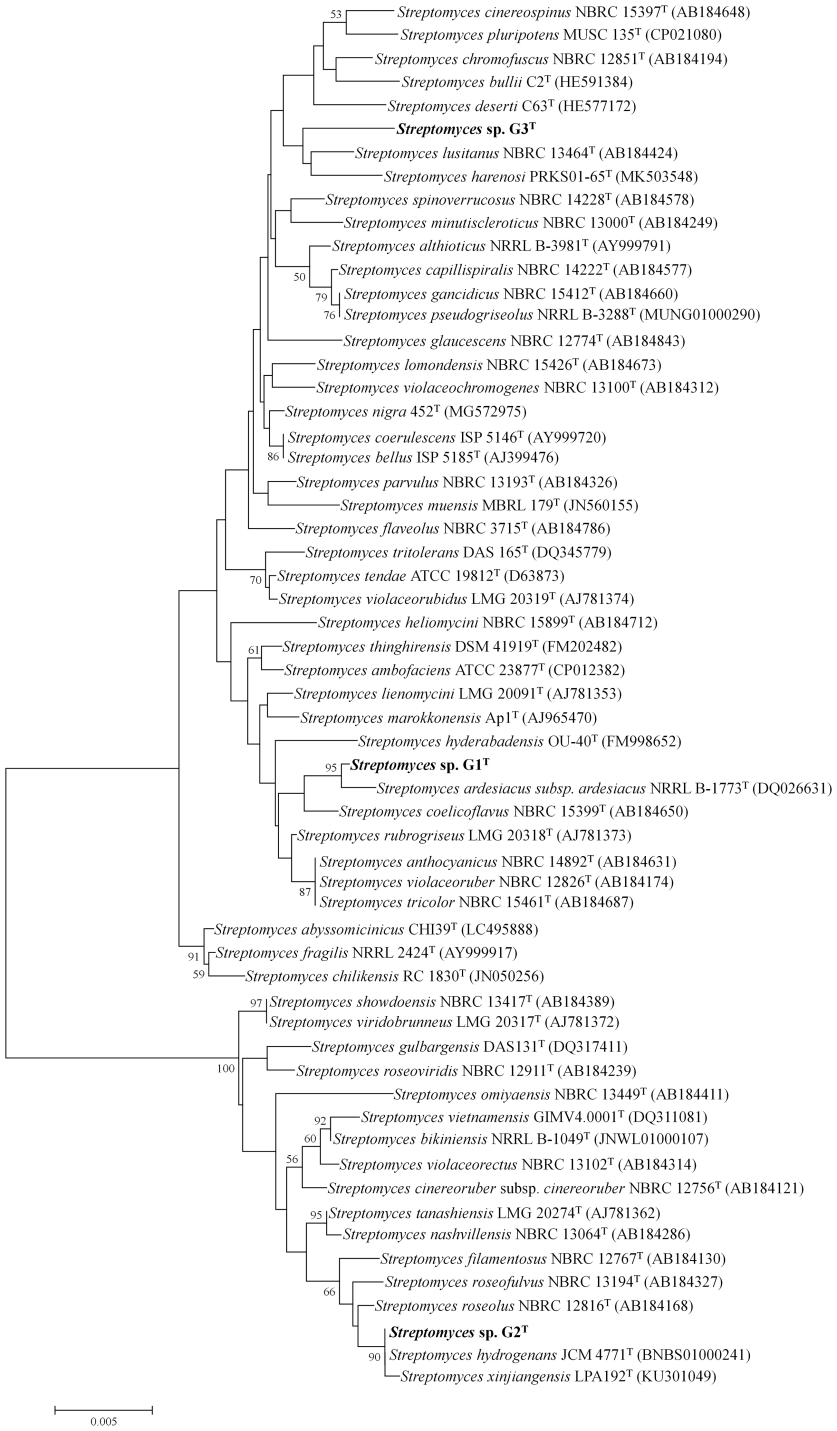
Neighbor-joining tree showing the phylogenetic position of species *Streptomyces* sp. G1^T^, *Streptomyces* sp. G2^T^, *Streptomyces* sp. G3^T^ and related taxa based on 16S rRNA gene sequences. Only bootstrap values above 50% (percentages of 1,000 replications) are indicated. Bar, 0.005 nucleotide substitutions per site.

The 16S rRNA gene sequences of G1–G3 were obtained from the whole-genome sequences (GenBank accession numbers JAMOZA000000000, JAMOZB000000000 and JAMOZC000000000, respectively). The phylogenetic tree based on the 16S rRNA gene sequences, constructed using MEGA 7.0, revealed that the three strains belonged to the genus *Streptomyces*. Strain G1 formed a distinct and stable branch with the nearest neighbor *S. diastaticus* subsp. *ardesiacus* NRRL B-1773^T^ (99.65% sequence similarity) supported by a high bootstrap value of 95% in the neighbor-joining tree (Figure 1). The neighbor-joining phylogenetic tree displayed that strain G2 produced a subclade with its closely related species *Streptomyces hydrogenans* JCM 4771^T^, *Streptomyces xinjiangensis* LPA192^T^ with 99.86 and 99.79% 16S rRNA gene sequence similarity (supported by a high bootstrap value of 90%). Distinguished with the above two isolates, G3 had a long evolutionary distance from other typical *Streptomyces* strains, with an independent and stable branch in the phylogenetic tree, and this clade clustered with *Streptomyces lusitanus* NBRC 13464^T^ (99.09%) and *Streptomyces harenosi* PRKS01-65^T^ (98.55%), which shared a low-grade bootstrap value below 50%. Additional information on similarity of organisms to the three straw-degrading species G1–G3 is listed in Supplementary Table S1. Moreover, to further assess the taxonomic status of the three strains, by comparison with local databases, a phylogenomic tree was constructed as described by Salam et al. (2020), based on 31 housekeeping genes: *dna*G, *frr*, *infC*, *nusA*, *pgk*, *pyr*G, *rplA*, *rplB*, *rplC*, *rplD*, *rplE*, *rplF*, *rplK*, *rplL*, *rplM*, *rplN*, *rplP*, *rplS*, *rplT*, *rpmA*, *rpoB*, *rpsB*, *rpsC*, *rpsE*, *rpsI*, *rpsJ*, *rpsK*, *rpsM*, *rpsS*, *smpB*, and *tsf* (Supplementary Figure S4). The neighbor-joining phylogeny of the most similar species at the species level was calculated using MEGA 7.0. Phylogenetic analysis according to the housekeeping genes showed that the three strains all belonged to the genus *Streptomyces*, and significantly differed from other similar strains, especially G1, which produced an individual and invariable subclade (Supplementary Figure S4A).

### Genome sequencing and bioinformatics analysis of G1–G3

**Figure 2:**
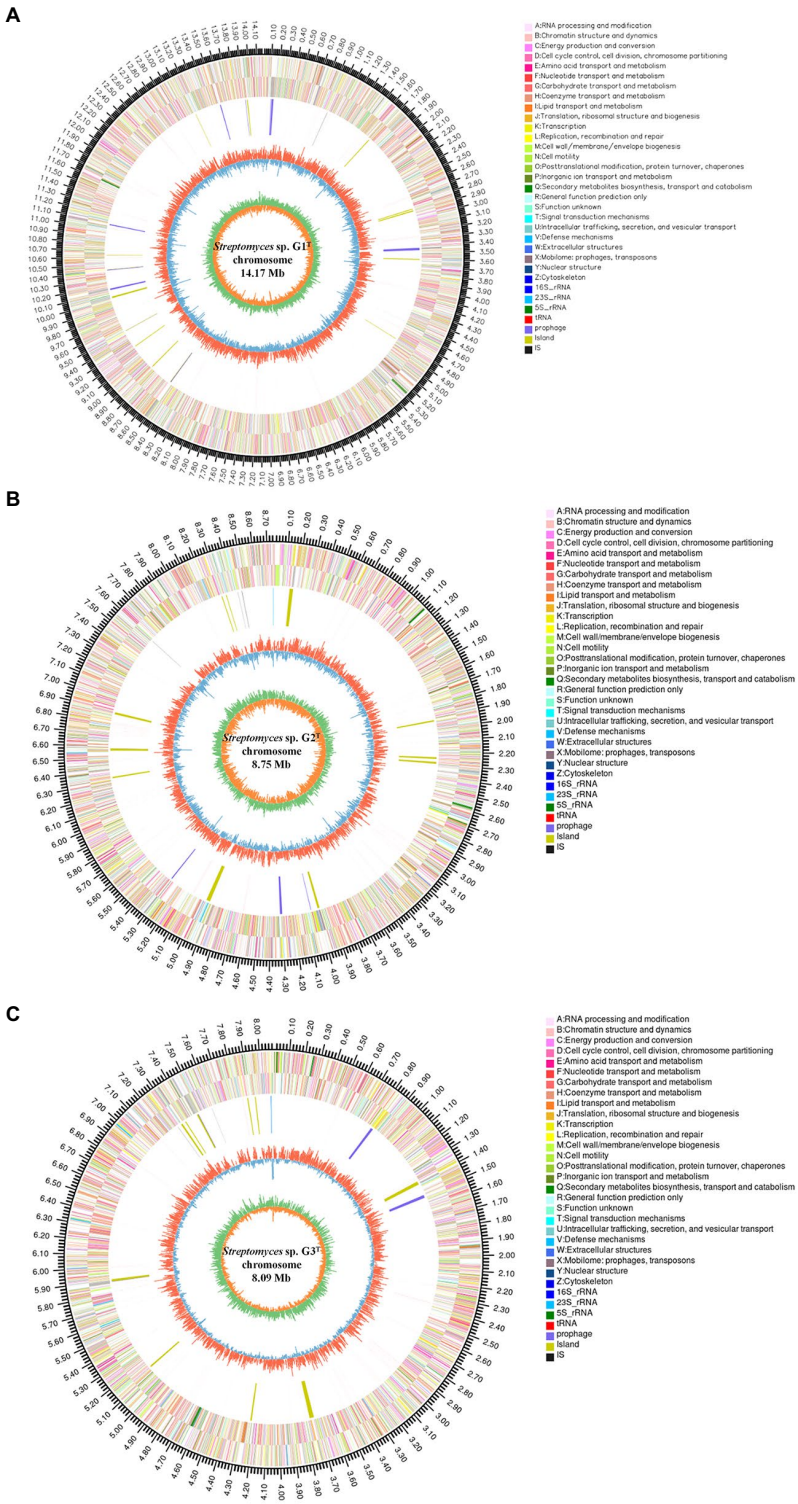
Genome map of the chromosome from strains *Streptomyces* sp. G1^T^ **(A)**, *Streptomyces* sp. G2^T^ **(B)** and *Streptomyces* sp. G3^T^ **(C)**. The circles for chromosome from the outside to the center represent the genome size, the CDS on the positive and negative strands with different colors indicating the different COG functional classifications, rRNA and tRNA, GC content, and GC skew, respectively.

**Figure 3:**
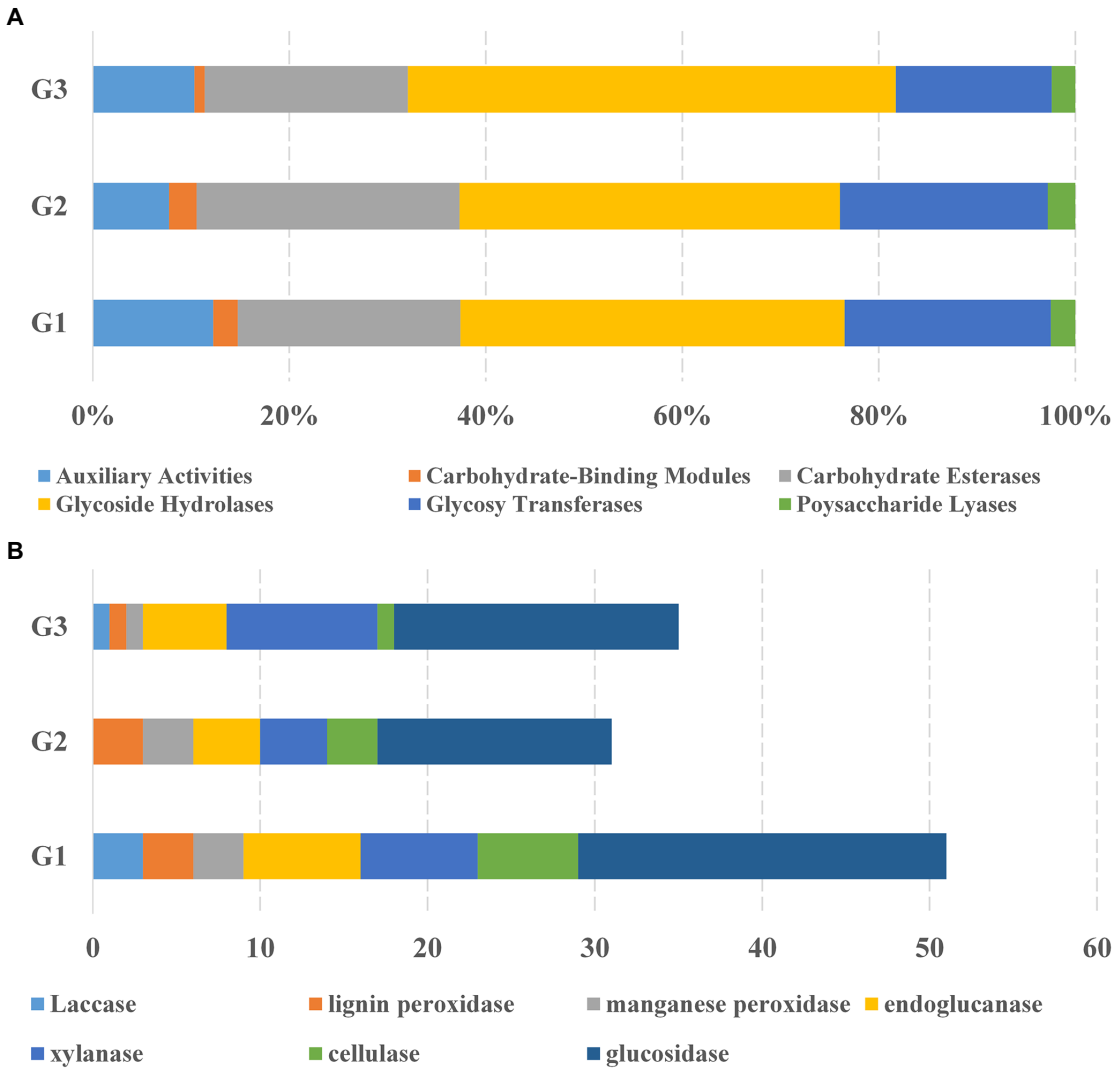
Comparative analysis of CAZyme of the species *Streptomyces* sp. G1^T^, *Streptomyces* sp. G2^T^ and *Streptomyces* sp. G3^T^. **(A)** The percentage of CAZymes in three biodegradable species distributed on six categories of enzyme activity: auxiliary activities, carbohydrate-binding molecules, carbohydrate esterases, glycoside hydrolases, glycosyltransferases, and polysaccharide lyases; **(B)** The total number of the CAZyme sets related to corn straw decomposition in the genomes of species *Streptomyces* sp. G1–G3.

To fully understand the molecular mechanisms of degradation of corn straw by strains G1–G3 and to explore their promising biological potential, the whole genomes of the three were sequenced and subsequently *de novo* assembled. The assembled genomes of G1–G3 were 14,173,392, 8,746,120 and 8,089,599 nucleotides in size, respectively, with corresponding G + C contents of 71.66, 73.34 and 72.21%. The circular chromosomes of samples, determined using the data visualizing software Circos, are presented in Figure 2. The general features of the genome sequences among the three strains are listed in Supplementary Table S2. Whole-genome shotgun sequences of G1–G3 were deposited at DDBJ/ENA/GenBank under accessions JAMOZA000000000, JAMOZB000000000 and JAMOZC000000000, respectively. Among these coding sequences, a total of 9,379, 5,915 and 5,584 genes in the G1–G3 genomes were classified into 24 clusters of orthologous groups of proteins (Figure 2). Most of the genes were related to functions like transcription, carbohydrate transport and metabolism, amino acid transport and metabolism, signal transduction mechanisms, coenzyme transport and metabolism, energy production and conversion, inorganic ion transport and metabolism, defense mechanisms, secondary metabolites biosynthesis, transport and catabolism. These functions are necessary for nutrient and antagonistic microbial competition, endowing the actinomycetes with competitive advantages in complex ecological environments. Interestingly, a similar phenomenon appeared in the test specimens based on the CAZyme annotation (Figure 3A). In the genomes of the three strains, there were a large number of genes encoding carbohydrate-related hydrolases, such as cellulase/endoglucanase (EC 3.2.1.4), glycosidase (EC 3.2.1.20/EC 3.2.1.21), xylanase (EC 3.2.1.8), laccase (EC 1.10.3.2), manganese peroxidase (EC 1.11.1.13) and lignin peroxidase (EC 1.11.1.14), which could effectively destroy the structure of lignocellulose in corn straw. CAZy annotation of the genome of species G1–G3 indicates differences in the number of genes coding for carbohydrate hydrolysis-related enzymes between them, as evidenced in Figure 3B. Strain G1 contains the largest number of genes encoding glycosidase, cellulase, xylanase and endoglucanase, which can effectively destroy the principal components of corn straw such as cellulose and hemicellulose. Genes belonging to cellulase, manganese peroxidase and lignin peroxidase are also enriched in species G2, and the number of encoded genes was higher than that of G3. In addition, compared with the isolate G2, strains G1 and G3 simultaneously possessed the genes encoding laccase, manganese peroxidase and lignin peroxidase, which played a crucial role in hydrolyzing the lignin. These results unambiguously confirmed the potential capacities of the three microorganisms to hydrolyze macromolecular polysaccharides at the molecular level. In addition, genes encoding chitinase/chitin deacetylase (EC 3.2.1.14 and EC 3.5.1.41), which function as a critical factor of biocontrol species in plasticizing the cell wall of phytopathogens (Langner and Göhre, 2016), were also distributed in the genomes of the three microorganisms. Chitin is an essential structural component in cell walls, and is the first line of microorganism defense in extreme environments or against other pathogens. Chitinase-generating organisms possibly have more competitive advantages in interactions with other pathogens in complex soil environments.

**Figure 4:**
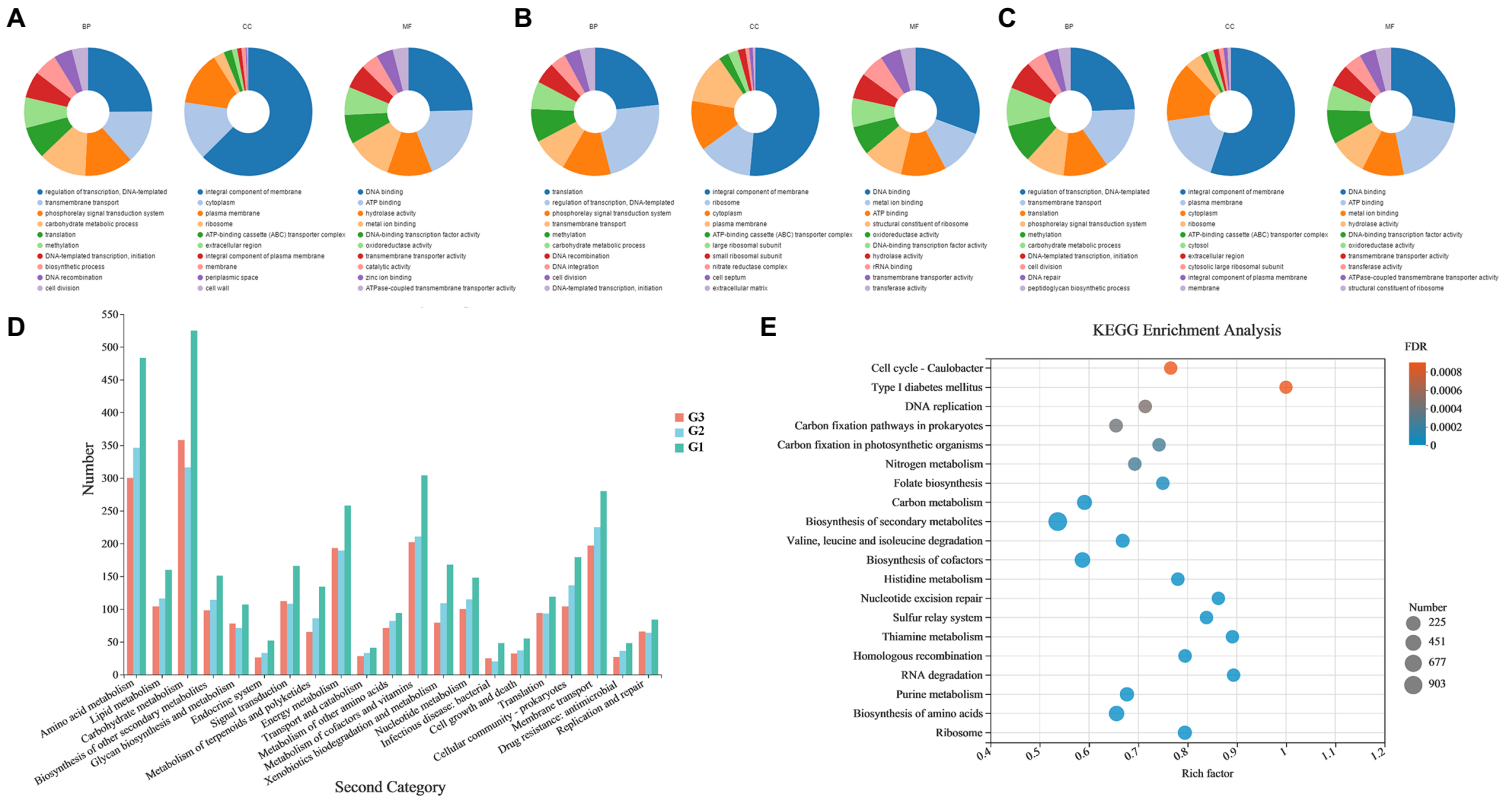
GO and KEGG functional annotations of *Streptomyces* sp. G1^T^, *Streptomyces* sp. G2^T^ and *Streptomyces* sp. G3^T^. **(A–C)** GO annotations of the three actinomyces with straw-decomposition ability; **(D)** Comparison of KEGG functional categories of the strains G1–G3; **(E)** KEGG enrichment bubble plot comparing *Streptomyces* sp. G1–G3. Vertical coordinate indicates the name of the pathways. Horizontal coordinate represents the enrichment rate. The dots’ size represents the number of genes in the pathway, and the color of the dots indicates the significance of the enrichment. Abbreviations: BP, biological process; CC, cellular component; MF, molecular function.

The GO and KEGG annotations were consistent with the above analysis results; genes encoding hydrolases related to carbon metabolism were widely distributed in the genomes of the three strains. For GO annotation (Figures 4A–C), G1–G3 had the largest number of genes annotated to molecular function (5,081, 695 and 2,299, respectively), followed by biological process and cellular component. In terms of biological processes, the gene content involved in the carbohydrate metabolic process (GO: 0005975) ranked in the top six annotated subcategories in each strain. According to the KEGG pathway annotation (Figure 4D), among the metabolic categories, amino acid and carbohydrate metabolisms were the two most abundant sub-branches of tested species, especially in strain G1. The result of KEGG enrichment bubble plot comparing G1–G3 also exhibited a similar phenomenon as above mentioned (Figure 4E). The most significant enrichment pathways include ‘biosynthesis of secondary metabolites’, “carbon metabolism’, “biosynthesis of cofactors”, etc. And also comprises the highest numbers coding genes in such pathway. Furthermore, a small number of genes related to biosynthesis of secondary metabolites was also analyzed. As described in reported studies, actinomycetes are a significant source of an important class of natural antibiotics. This property allows species to inhibit pathogenic bacteria and fungi present in the soil, thus maintaining their normal function while protecting plants from the invasion of pathogenic microorganisms.

### Impacts of different treatments on corn straw degradation rate

In order to better identify the degrading capacity of microbial consortium G123 on corn straw, three degrading treatment groups and processing time gradients (incubation of about 11 and 17 weeks) were set up in field experiments. The dynamics of the corn straw degradation rate are presented in Table 3. The degradation rate dramatically improved as the processing time extended. The fastest degradation rate of corn straw was clearly for the SCG, followed by SCW and SC treatments, at both of the time points. The degradation degree of the straw was also evidently enhanced with the prolongation of treatment time. The above experimental results strongly suggest that the screened actinomycetes secreted abundant and high-efficiency biodegradation enzymes, which participated in the plant lignocellulose degradation, resulting in significant disruption to the structure of macromolecular polysaccharides in the straw. The SCG treatment performed well in the field experiment, not only due to better release of hydrolases by microbial consortium G123 thus destroying the fiber structure of the straw, but also possibly related to the extensive environmental adaptability of the actinomycetes. Previous studies showed that actinomycetes have some resistance to alkaline, high and low temperature and other extreme environments (Shao et al., 2015).

**Table 3:** The decomposition rate of cornstalk under different treatments.

Time (days)	Treatment groups	Decomposition rate (%)
77	SC-F-I	39.10 ± 0.20a
	SCW-F-I	44.27 ± 0.40b
	SCG-F-I	51.60 ± 0.26c
119	SC-F-II	42.53 ± 0.49a
	SCW-F-II	55.50 ± 0.40b
	SCG-F-II	66.37 ± 0.25c

### Content changes of corn straw after degradation

Biological, chemical or physical pretreatment of straw is usually used to separate polymers and expose soluble components (Mussoline et al., 2013; Wang Z. C. et al., 2020). With time passing, the dominant components of the corn straw constantly varied during the degradation process. The changes of residual content of corn straw after degradation with various treatments differed remarkably, but shared the same general tendency (Table 4). The residual cellulose content of corn straw in each specimen showed a trend of initial increase and then decrease. After 11 weeks of degradation, the mass percentages of residual cellulose in SC-F, SCW-F and SCG-F significantly rose to 39.10, 36.52 and 35.32%, respectively, from the initial 31.99%; these correspondingly decreased to 27.99, 27.64 and 24.55% after 17 weeks of digestion. The mass fraction of the remaining hemicellulose in samples gradually decreased with increased incubation time, and the values of the treatment groups were significantly lower than the initial percentage, especially for SCG-F, which utilized the microbial consortium G123. The percentage of residual lignin in corn straw degraded by different groups was higher than the initial value at both time points. During the digestion process, the relative content of cellulose and hemicellulose decreased significantly, and the degradation rate of hemicellulose was faster than that of cellulose. Compared with other treatments, the content of residual cellulose and hemicellulose was lower in SCG-F, which might have promoted the degradation of hemicellulose and cellulose though secreting high-efficiency hydrolases.

**Table 4:** Cellulose, hemicellulose and lignin content of corn straw samples.

Treatments	Cellulose (%)	Hemicellulose (%)	Lignin (%)
C	31.99 ± 0.15	25.33 ± 0.53	21.17 ± 0.13
SC-I	39.10 ± 0.59a	17.13 ± 0.13b	27.08 ± 0.28b
SCW-I	36.52 ± 0.66b	17.94 ± 0.21a	28.96 ± 0.20a
SCG-I	35.32 ± 0.67b	17.15 ± 0.37b	27.27 ± 0.22b
SC-II	27.99 ± 0.51a	12.22 ± 0.27a	27.73 ± 0.46c
SCW-II	27.64 ± 0.01a	11.85 ± 0.04b	32.58 ± 0.18b
SCG-II	24.55 ± 0.39b	10.18 ± 0.16c	36.07 ± 0.07a

### Corn straw morphology after digestion treatments

The outer surface of untreated corn straw showed a waxy layer with a smooth, compact and seamless surface (Supplementary Figure S5A). This indicated that the straw sample retained intact structure before treatment, and its dense surface structure was the primary hindrance to the normal breakdown of cellulose, affecting its hydrolysis. When the straw samples were imbedded in soil for 17 weeks, the outer epidermis of corn straw was destroyed, and the surface become rough and cracked with slight porosity (Supplementary Figure S5B). The internal structure of SCW-F-treated corn straw was irregular (Supplementary Figure S5C), exhibiting a rough and fragmented surface with large amounts of small voids. In contrast, the structure of SCG-F-treated corn straw was seriously damaged (Supplementary Figure S5D). Based on these observations, we propose that the individual degradation agents destroyed some of the recalcitrant structure of straw, whereas the microbial consortium G123 functioned more efficiently.

### Structural analysis by FTIR

**Figure 5:**
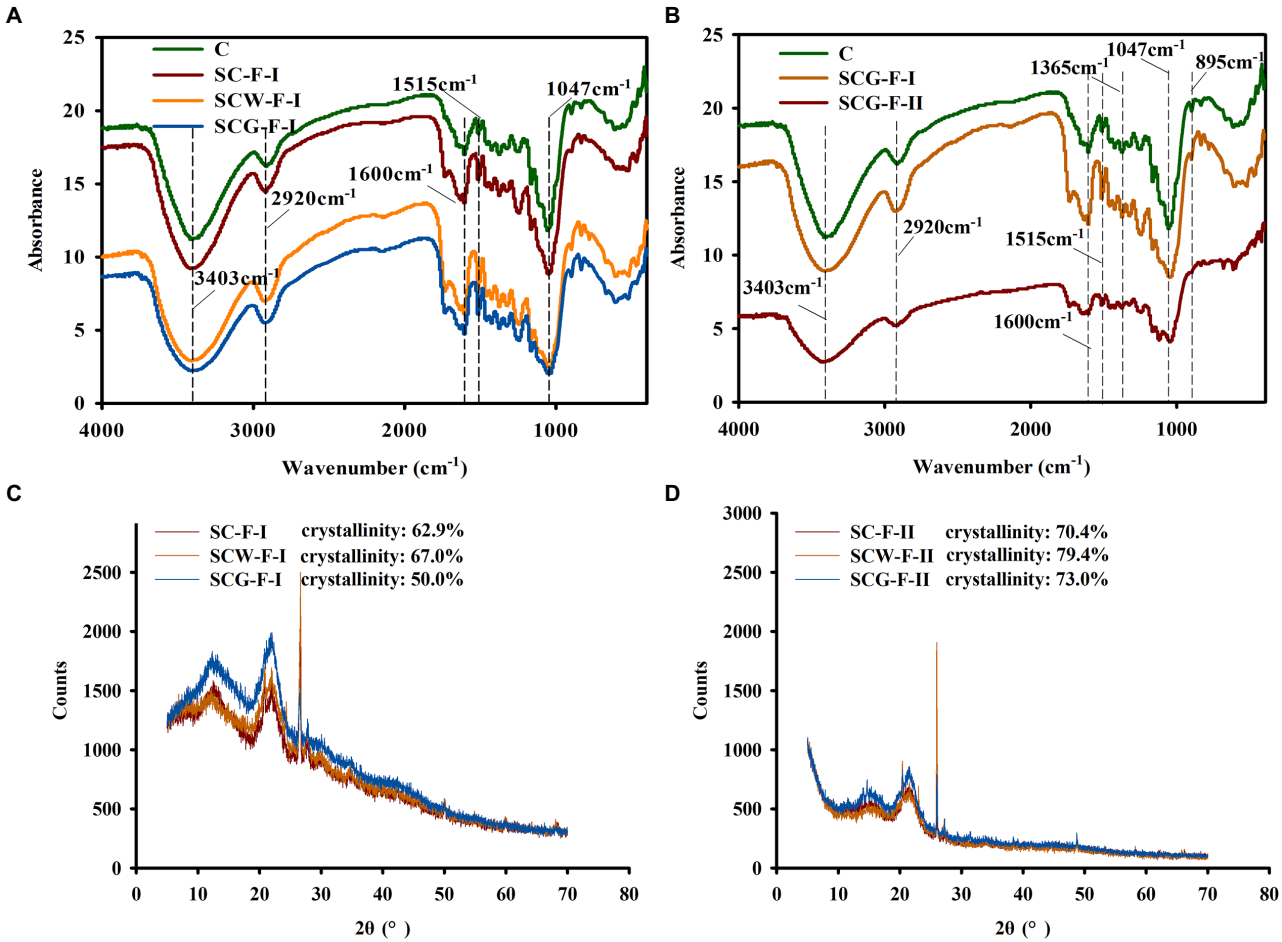
Fourier transform-infrared spectroscopy (FT-IR) and X-ray diffraction analysis of different corn straw samples. **(A)** Infrared spectrum analysis of cornstalk samples after treatment with straw-degradation agent and microbial consortium G123 for 11 weeks; **(B)** Variations in functional groups of corn straw samples after treatment with microbial consortium G123 for 11 and 17 weeks. **(C)** X-ray diffraction pattern of cornstalk samples after treating with 11 weeks; **(D)** X-ray diffraction pattern of cornstalk samples after treating with 17 weeks.

To thoroughly understand the function of microflora in changing the structure and properties of corn straw, the chemical variations of straw samples were analyzed by FTIR (Figure 5A). There were many absorption peaks in the infrared spectrum range, reflecting the content and composition of cellulose and lignin in samples. The absorption band at 3300–3500 cm^−1^ is representative of O–H intermolecular vibrations and caused by O–H stretching in cellulose. The peak of SCG-F was sharply reduced at 3300–3500 cm^−1^ (Figure 5A), indicating that degrading microbes could break the hydrogen bonds between cellulose molecules more efficiently, consistent with the variation in cellulose content (Table 4). The peak of the SCG-F treatment group increased at 1053 cm^−1^ and decreased at 1058 cm^−1^, indicating that cellulose and hemicellulose were degraded and transformed. The characteristic peaks of untreated lignin are at 1515 and 1,650 cm^−1^ (Salehian et al., 2013). The spectral peak at 1515 cm^−1^ slightly changed, suggesting that only a small amount of the aromatic rings were degraded in straw, consistent with the change in lignin content (Table 4). Cellulose consists of *β*-D-glucopyranosyl monomers interconnected by (1–4)-*β*-glycoside bonds, which have a characteristic absorption peak at 895 cm^−1^, to generate the linear polymer structure. The absorption peak at 1365 cm^−1^ is related to the bending vibration of the C–H group of lignin. The characteristic peaks around 1,365, 1,600 and 1,500 cm^−1^ significantly declined for the G123 treatment at the later stage of incubation (Figure 5B), indicating serious damage to lignin structure following decomposition by G123.

### XRD analysis

For the study of fiber structure, the theory of two-phase structure is generally adopted the microstructure of fiber is considered to be composed of crystalline and amorphous regions. The crystallinity is an important index to measure the properties of cellulose, and reflects the degree of crystallinity in the process of cellulose aggregation (Liu, 2004). After degradation by different treatment groups, the structure and crystallinity of samples changed, due to removal of major components from the corn straw. The spectrum had dominant and secondary peaks at 22.0° and 12°-15.0°, respectively, corresponding to the diffraction intensity of the crystalline and amorphous regions. The crystallinity of each treatment increased with longer treatment time (Figures 5C,D), likely for two primary reasons. Firstly, biodegradation microorganisms removed more hemicellulose components, thus increasing the cellulose content and so greatly improving overall crystallinity, consistent with the results in Table 4. Secondly, the microbial consortium G123 might preferentially impact the disordered non-crystalline region of cellulose in corn straw at a higher rate than the crystalline region. In addition, the sample digested with microbial consortium G123 had the lowest crystallinity (50%), likely due to faster decomposition rate of cellulase acting on the crystalline region than the other treatments. The treatment group SCG-II had higher crystallinity than the SCG-I, and crystallinity was significantly higher for SCG than the other two groups. The degrading enzymes primarily hydrolyzed the non-crystalline region of corn straw, and then penetrated into the crystalline region (Table 4). It could also be inferred that the degradation degree of microbial consortium G123 was higher for the non-crystalline than the crystalline area, consistent with the results of Gong et al. (2020).

### Bacterial community changes in soil from different treatment groups

**Figure 6:**
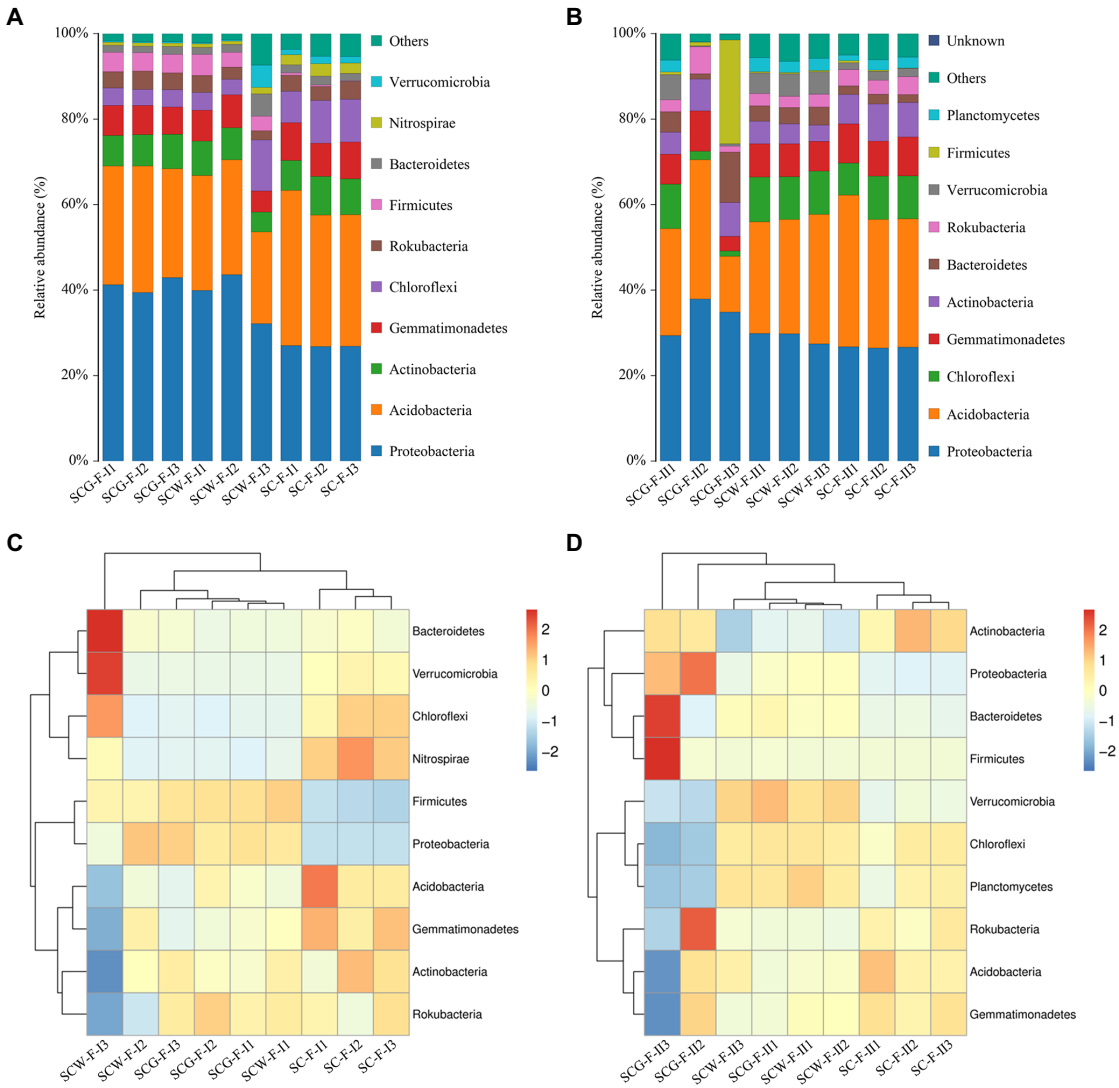
Taxonomic classification and relative abundance of bacterial communities at phylum level: **(A,C)** sampled after 11 weeks cultivation; **(B,D)** sampled after 17 weeks cultivation.

The high-throughput sequencing and high-quality sequencing reads, at 97% sequence similarity degree with a confidence threshold of 0.005% in various specimens, gave ranges of 741–1,055 (11 weeks, BioProject accession number is PRJNA888828) and 965–2,134 (17 weeks, BioProject accession number is PRJNA888837) OTUs. In addition, there were evident discrepancies in bacteria at the phyla level among the treatments (Figures 6A,C). At the phylum level, *Proteobacteria* (32.10–42.88%) were the most abundant microorganisms in both SCW-F and SCG-F, whereas it was *Acidobacteria* (30.73–36.33%) in the control group SC-F (Figure 6A). Other dominant bacterial phyla in the three treatment groups were *Actinobacteria* (4.61–9.03%), *Gemmatimonadetes* (4.97–8.81%) and *Chloroflexi* (3.60–11.92%). In the field experiments, the average relative abundances of *Proteobacteria* and *Firmicutes* were improved by straw returning though supplementing the additional degradation flora G123 compared to the other two treatments. However, application of the degradation actinomycetes led to a marked reduction in amounts of *Acidobacteria*, *Gemmatimonadetes*, *Chloroflexi*, *Nitrospirae* and *Verrucomicrobia*. These results revealed that the bacterial diversity was directly related to the straw returning and addition of decomposing agent and *Streptomyces* spp. For the purpose of improving our understanding of the impacts of the additional decomposing actinomycetes on microorganism diversity in soil after straw returning, the community changes of the samples treated for 17 weeks were analyzed by high-throughput sequencing (Figures 6B,D). The results were the same as for 11 weeks of incubation, distinguished from the reference group in which the most abundant phylum was *Acidobacteria* (29.99–35.50%), with *Proteobacteria* accounting for the highest percentage of the microbial population in the SCW-F (27.41–29.80%) and SCG-F treatments (29.32–37.86%), and followed by *Chloroflexi*, *Gemmatimonadetes* and *Actinobacteria*. Compared with SC-F and SCW-F treatments, abundances of *Proteobacteria*, *Bacteroidetes* and *Firmicutes* were significantly enhanced with straw returning, whereas *Acidobacteria*, *Chloroflexi* and *Planctomycetes* levels were reduced. The data of disparate specimens showed that longer treatment time resulted in significant increases in abundance of *Chloroflexi*, *Bacteroidetes*, *Firmicutes* and *Gemmatimonadetes*, and the microbial diversity of each treatment was also dramatically improved, suggesting that corn straw returning influenced the composition and abundance of soil communities.

Furthermore, at the order level, the microbiome composition was dominated by *Betaproteobacteriales*, *Gemmatimonadales*, *Pyrinomonadales*, *Rhizobiales*, and *Myxococcales*. Compared with the SC-F group, the relative abundances of *Betaproteobacteriales*, which had the highest percentage in all samples, were obviously enhanced in SCW-F and SCG-F, whereas the increasing trend was significantly prevented in SCW-F after 17 weeks (Supplementary Figure S6A). *Rhizobiales* and *Myxococcales* were also dramatically higher in both SCW-F and SCG-F compared the reference group SC-F, especially after 11 weeks of treatment (Supplementary Figures S6B,C). Moreover, a similar phenomenon was also observed at the order level following the supplementation of microbial consortium G123, the relative abundances of *Gammaproteobacteria* (Supplementary Figure S6D) and *Alphaproteobacteria* (Supplementary Figure S6E) revealed an increasing tendency and was also the highest among the three treatment groups, especially at the first sampling time point. These consequences indicated that the additional decomposing actinomycetes G123 had a significant effect on both the abundance and composition structure of soil microbial flora.

## Discussion

*Streptomyces*, which was the most representative genus of the phylum *Actinobacteria*, is an important source of versatile promising metabolites and carbohydrate-related hydrolases (Prakash et al., 2013; Sharma et al., 2016; He et al., 2018). Actinomycetes are a transition group between bacteria and fungi in the degree of evolution, with plentiful vegetative hyphae and aerial hyphae generated that can differentiate to produce terrific numbers of spores. The presence of spores in *Streptomyces* spp. gives them a natural advantage under selective pressure, which might be associated with the need to survive outside of plants, in soil or in other extreme environments (Sivalingam et al., 2019). Their extensive distribution in terrestrial ecosystems is another reason why this genus has received much attention. In previous research, many *Streptomyces* species with the ability to produce extracellular hydrolase have been screened and identified. *Streptomyces griseorubens* C-5, with the capacity to simultaneously produce cellulase, laccase, peroxidase, xylanase and pectinase, was separated from humus-rich soil. These hydrolases can effectively destroy the structure of rice straw, and SEM showed that the biodegradation process of C-5 could accelerate decomposition by colonizing the inner tissues of rice straw through generating mycelial pellets (Xu and Yang, 2010). *Streptomyces griseorubens* JSD-1 has potential lignocellulose-degrading activity and phytopathogenic fungal resistance, and was isolated from soil and rotten straw sampled under stooks stacked for several years (Feng et al., 2013). Evidently increased carbohydrate/lignin ratio of wheat straw was observed after digestion by *S. viridosporus* T7A, indicating efficient degradation of lignin, which is one of the major contributing factors to stress resistance of lignocellulosic biomass (Zeng et al., 2013). Therefore, mining novel *Streptomyces* resources or constructing highly efficient decomposing bacteria consortia are of great significance for industrial and agricultural production.

As the world’s largest grain producer, China produced approximately 700 million tons of grain in 2021 (http://www.stats.gov.cn/English./PressRelease/202112/t20211207_1825086.html). In addition, total straw production accounts for nearly 40% of the total biomass output. The continuous improvement of crop yields has resulted in a large amount of straw residue output annually. Corn straw is mainly composed of cellulose, hemicellulose and lignin, and is an important and abundant carbohydrate resource in industrial and agricultural production. However, much corn straw is disposed of improperly or burned directly, leading to great resource waste and serious threats to the environment. Therefore, rational utilization of straw resources is essential for alleviating energy and environmental pressure. Directly returning the straw to fields is often considered to increase phytopathogen loads and improve morbidity or disease indexes (Qiao et al., 2013). To facilitate the utilization of carbohydrates in lignocellulosic biomass, biodegradation basically overcomes the physical and chemical barriers of lignocellulosic complexes, and enhances antagonism against pathogens. With the aim of accelerating the reutilization of corn straw, our research group screened straw biodegradation strains G1–G3, which clearly showed cellulose, hemicellulose and lignin degradation activities, from the humus of straw returned to the field for many years in the cold region of China (Gong et al., 2020). Construction of the *in situ* microbial consortium G123 for corn straw, and optimizing the fermentation conditions resulted in a significantly improved weight loss rate of straw. To improve our understanding of these degrading strains, their taxonomy and morphological, physiological, biochemical and chemotaxonomic characteristics were analyzed, clearly revealing that the three strains had typical characteristics of *Streptomyces* and might be novel taxa of this genus. Genome sequencing, recognized as an effective method for studying functions and bioinformatic analysis of genomic features such as the whole genome structure and the presence or absence of specific genes, could help in understanding the decomposition mechanism of corn straw at the molecular level, which paves the way for developing biotechnological utilization of these species. The G1–G3 genomes contained massive numbers of genes related to carbohydrate metabolism, among which the most important are those encoding carbohydrate hydrolases (Figures 3, 4). These hydrolases, including cellulase/endoglucanase, glycosidase, xylanase, laccase, manganese peroxidase and lignin peroxidase, all significantly contribute to the degradation of lignocellulose in corn straw. The potential of these three microorganisms to hydrolyze macromolecular polysaccharides was unambiguously illustrated at the molecular level. Furthermore, corn straw-returning experiments were used to evaluate the application efficiency of the microbial consortium G123. The G123 showed a higher decomposing rate of straw compared with the commercial degradation agent (Table 3), with a significant increasing tendency with longer treatment time. In degrading straw samples, SEM was employed to detect the degradation ability of decomposition microbes. The SCW and SCG treatments seriously damaged the corn straw structure, and the internal structure of straw became irregular, rough and fragmented with large numbers of voids **(** Supplementary Figures S5C,D). This indicated that the degradation agent could destroy some of the recalcitrant structure of straw, whereas microbial consortium G123 functioned more efficiently. The FTIR and XRD analysis was further used to assess the variations in straw structure. The peak of SCG-F was dramatically reduced at 3300–3500 cm^−1^ (Figure 5A), which is representative of O–H vibrations due to O–H stretching in cellulose, indicating that G123 could efficiently destroy the connecting bonds between cellulose molecules. The destruction of chemical bonds could transform the cellulose structure of straw, enhance the accessibility of the substrate, facilitating the degradation of cellulose (Shi et al., 2018). In the latter phase of the field trial, the characteristic peaks of lignin were markedly reduced (Salehian et al., 2013), indicating that lignin structure was seriously damaged after decomposition by G123 (Figure 5B). The XRD analysis also showed that the additions of degrading bacteria could effectively break the main active components of straw. The crystallinity of each treatment increased with longer treatment time (Figures 5C,D), possibly indicating that the cellulase secreted by G123 had a greater rate of straw hydrolysis in the non-crystalline compared to the crystalline region. In addition, environment factors, especially temperature, have significant effects on microbial community associated with straw degradation (Zhou et al., 2015). In a certain range, with the increase of culture temperature, activity of straw degradation microorganisms increases, which can accelerate straw degradation (Feng and Simpson, 2009). However, temperature is always low in the northeastern region of China, which brings great challenges to our research and remains one of the main factors to be overcome.

The composition of microbial communities is an important basis for the function performed by microorganisms (Torsvik et al., 2002). For this purpose, microbial diversity and composition under decomposition by different treatment groups were determined using Illumina MiSeq sequencing at the two sampling time points. Similar bacterial communities were found in the three treatment groups at the phyla level: *Proteobacteria* and *Acidobacteria* were the top two phyla followed by *Actinobacteria*, *Gemmatimonadetes* and *Chloroflexi* (Figure 6A), which roughly corresponded to reported findings (Huang et al., 2021). It speculated that the influence of the microbial diversity variation between the commercial reagent and microbial consortium G123 is exceptionally similar, however, there were several differences in the average relative content of certain bacteria. Compared with SC-F and SCW-F, the average relative abundances of *Proteobacteria* and *Firmicutes* were higher when supplemented with microbial consortium G123. Furthermore, the data of disparate specimens illustrated that longer treatment time significantly enhanced the abundance of *Chloroflexi*, *Bacteroidetes*, *Firmicutes* and *Gemmatimonadetes*, and the microbial diversity of each treatment was also dramatically improved (Figure 6B), suggesting that corn straw returning influenced the composition and abundance of soil communities. *Proteobacteria*, *Actinobacteriota* and *Actinobacteria*, as the main constituents in agricultural soil, contribute to decomposing organic matter and colonizing nutrient-rich environments (Liang et al., 2020). Other reports suggest that the higher levels of *Proteobacteria* may improve soil fertility, plant growth and protection against diseases (Chaudhry et al., 2012; Yang et al., 2018). Major bacterial communities of *Proteobacteria*, *Acidobacteria*, *Bacteroidetes* and *Firmicutes* have been reported to be associated with soil antibiotic resistance and antibiotic degradation (Islas-Espinoza et al., 2012; Caban et al., 2018). In addition, at the order level, in comparison with the control group SC-F, the relative abundances of *Betaproteobacteriales* and *Rhizobiales* showed a significant increasing trend (Supplementary Figures S6A,B). Most microorganisms of these orders are commonly described as diazotrophs (Im et al., 2006). Members of *Myxococcales* are popularized as “wolfpack” predators that can not only survive under oligotrophic conditions and other extreme environments, but also produce multiple antibacterial molecules to inhibit growth of antagonistic microbes (Wang W. H. et al., 2020). The increased content of such microorganisms improves the adaptability of microflora to the environment (Supplementary Figure S6C). Moreover, microbes of *Gammaproteobacteria*, which are attractive candidates for their applications in biological control of plant diseases and nematodes (Hayward et al., 2010), were also dramatically improved in SCG-F compared with SC-F and SCW-F (Supplementary Figure S6D). After application of microbial consortium G123, the abundance and diversity of soil microorganisms changed dramatically, these variations might offer excellent nutrients for subsequent plant growth, as well as better resistance to pathogenic strains.

The present work described three straw degradation strains, which might be novel *Streptomyces* taxa, and evaluated the application efficiency of microbial consortium G123 in straw-returning experiments. This study promotes understanding of the straw degradation mechanism at the molecular level, provides a scientific basis to explain the variation tendency of soil microorganisms after straw returning to the field and offers promising insights into managing corn straw resources.

## Data availability statement

The datasets presented in this study can be found in online repositories. The names of the repository/repositories and accession number(s) can be found in the article/Supplementary material.

## Author contributions

XG and CQ contributed to the concept and design. YY, YH, and QW performed the research and analyzed the data. JM, YJ, GL, and LL wrote the manuscript. All authors contributed to manuscript revision and read and approved the submitted version.

## Funding

This research was supported by China Agriculture Research System of MOF and MARA (Ministry of Finance and Ministry of agriculture and rural areas), Research and demonstration of key technologies for obstacle reduction and capacity improvement in Albic soil areas of the Sanjiang Plant (Number of assignment: 2022YFD1500800). Agricultural Science and Technology Innovation Spans Project of the Heilongjiang Academy of Agricultural Sciences (HNK2019CX12) and the Strategic Priority Research Program of the Chinese Academy of Sciences (XDA28030403-7).

## Conflict of interest

The authors declare that the research was conducted in the absence of any commercial or financial relationships that could be construed as a potential conflict of interest.

## Publisher’s note

All claims expressed in this article are solely those of the authors and do not necessarily represent those of their affiliated organizations, or those of the publisher, the editors and the reviewers. Any product that may be evaluated in this article, or claim that may be made by its manufacturer, is not guaranteed or endorsed by the publisher.

## Footnotes

^1^ http://soap.genomics.org.cn/soapdenovo.html
