# Supplementary material for: Characterizing corn-straw-degrading actinomycetes and evaluating application efficiency in straw-returning experiments
Source: Front Microbiol. 2022 Dec 5;13:1003157. doi: 10.3389/fmicb.2022.1003157 (PMC9760696; doi:10.3389/fmicb.2022.1003157)
Supplement: Supplementary file 1 [file Table_1.DOCX]

**Supplementary Table S1.** Similarity species of cornstalk-decomposition species G1–G3.

| Strains number | Similar strains | Accession number | 16S rRNA similarity rate (%) |
| --- | --- | --- | --- |
| G1 | *Streptomyces ardesiacus* subsp. ardesiacus NRRL B-1773^T^ | DQ026631 | 99.65 |
| G1 | *Streptomyces coelicoflavus* NBRC 15399^T^ | AB184650 | 99.65 |
| G1 | *Streptomyces rubrogriseus* LMG 20318^T^ | AJ781373 | 99.17 |
| G1 | *Streptomyces fragilis* NRRL 2424^T^ | AY999917 | 99.03 |
| G1 | *Streptomyces thinghirensis* DSM 41919^T^ | FM202482 | 98.96 |
| G1 | *Streptomyces abyssomicinicus* CHI39^T^ | LC495888 | 98.96 |
| G1 | *Streptomyces anthocyanicus* NBRC 14892^T^ | AB184631 | 98.96 |
| G1 | *Streptomyces violaceoruber* NBRC 12826^T^ | AB184174 | 98.96 |
| G1 | *Streptomyces lienomycini* LMG 20091^T^ | AJ781353 | 98.96 |
| G1 | *Streptomyces tricolor* NBRC 15461^T^ | AB184687 | 98.95 |
| G1 | *Streptomyces marokkonensis* Ap1^T^ | AJ965470 | 98.89 |
| G1 | *Streptomyces chilikensis* RC 1830^T^ | JN050256 | 98.89 |
| G1 | *Streptomyces ambofaciens* ATCC 23877^T^ | CP012382 | 98.83 |
| G1 | *Streptomyces tendae* ATCC 19812^T^ | D63873 | 98.76 |
| G1 | *Streptomyces violaceorubidus* LMG 20319^T^ | AJ781374 | 98.76 |
| G1 | *Streptomyces heliomycini* NBRC 15899^T^ | AB184712 | 98.76 |
| G1 | *Streptomyces tritolerans* DAS 165^T^ | DQ345779 | 98.69 |
| G1 | *Streptomyces hyderabadensis* OU-40^T^ | FM998652 | 98.64 |
| G1 | *Streptomyces althioticus* NRRL B-3981^T^ | AY999791 | 98.61 |
| G1 | *Streptomyces flaveolus* NBRC 3715^T^ | AB184786 | 98.55 |
| G2 | *Streptomyces hydrogenans* JCM 4771^T^ | BNBS01000241 | 99.86 |
| G2 | *Streptomyces xinjiangensis* LPA192^T^ | KU301049 | 99.79 |
| G2 | *Streptomyces tanashiensis* LMG 20274^T^ | AJ781362 | 99.31 |
| G2 | *Streptomyces nashvillensis* NBRC 13064^T^ | AB184286 | 99.31 |
| G2 | *Streptomyces gulbargensis* DAS131^T^ | DQ317411 | 99.31 |
| G2 | *Streptomyces roseolus* NBRC 12816^T^ | AB184168 | 99.3 |
| G2 | *Streptomyces filamentosus* NBRC 12767^T^ | AB184130 | 99.17 |
| G2 | *Streptomyces roseoviridis* NBRC 12911^T^ | AB184239 | 99.16 |
| G2 | *Streptomyces violaceorectus* NBRC 13102^T^ | AB184314 | 98.89 |
| G2 | *Streptomyces cinereoruber* subsp. *cinereoruber* NBRC 12756^T^ | AB184121 | 98.82 |
| G2 | *Streptomyces showdoensis* NBRC 13417^T^ | AB184389 | 98.82 |
| G2 | *Streptomyces roseofulvus* NBRC 13194^T^ | AB184327 | 98.82 |
| G2 | *Streptomyces viridobrunneus* LMG 20317^T^ | AJ781372 | 98.69 |
| G2 | *Streptomyces vietnamensis* GIMV4.0001^T^ | DQ311081 | 98.66 |
| G2 | *Streptomyces bikiniensis* NRRL B-1049^T^ | JNWL01000107 | 98.62 |
| G2 | *Streptomyces omiyaensis* NBRC 13449^T^ | AB184411 | 98.54 |
| G3 | *Streptomyces lusitanus* NBRC 13464^T^ | AB184424 | 99.09 |
| G3 | *Streptomyces spinoverrucosus* NBRC 14228^T^ | AB184578 | 98.89 |
| G3 | *Streptomyces lomondensis* NBRC 15426^T^ | AB184673 | 98.89 |
| G3 | *Streptomyces nigra* 452^T^ | MG572975 | 98.76 |
| G3 | *Streptomyces coerulescens* ISP 5146^T^ | AY999720 | 98.73 |
| G3 | *Streptomyces minutiscleroticus* NBRC 13000^T^ | AB184249 | 98.69 |
| G3 | *Streptomyces gancidicus* NBRC 15412^T^ | AB184660 | 98.68 |
| G3 | *Streptomyces bellus* ISP 5185^T^ | AJ399476 | 98.66 |
| G3 | *Streptomyces pseudogriseolus* NRRL B-3288^T^ | MUNG01000290 | 98.62 |
| G3 | *Streptomyces cinereospinus* NBRC 15397^T^ | AB184648 | 98.62 |
| G3 | *Streptomyces chromofuscus* NBRC 12851^T^ | AB184194 | 98.61 |
| G3 | *Streptomyces althioticus* NRRL B-3981^T^ | AY999791 | 98.61 |
| G3 | *Streptomyces bullii* C2^T^ | HE591384 | 98.61 |
| G3 | *Streptomyces deserti* C63^T^ | HE577172 | 98.59 |
| G3 | *Streptomyces pluripotens* MUSC 135^T^ | CP021080 | 98.55 |
| G3 | *Streptomyces harenosi* PRKS01-65^T^ | MK503548 | 98.55 |
| G3 | *Streptomyces parvulus* NBRC 13193^T^ | AB184326 | 98.55 |
| G3 | *Streptomyces glaucescens* NBRC 12774^T^ | AB184843 | 98.55 |
| G3 | *Streptomyces capillispiralis* NBRC 14222^T^ | AB184577 | 98.54 |
| G3 | *Streptomyces violaceochromogenes* NBRC 13100^T^ | AB184312 | 98.54 |
| G3 | *Streptomyces muensis* MBRL 179^T^ | JN560155 | 98.54 |
