# Supplementary material for: Characterizing corn-straw-degrading actinomycetes and evaluating application efficiency in straw-returning experiments
Source: Front Microbiol. 2022 Dec 5;13:1003157. doi: 10.3389/fmicb.2022.1003157 (PMC9760696; doi:10.3389/fmicb.2022.1003157)
Supplement: Supplementary file 2 [file Table_2.DOCX]

**Supplementary Table S2.** Comparative genomic features among *Streptomyces* sp. G1–G3.

| **Organism** | **Accession No** | **Sequencing Technology** | **Genome coverage** | **Genome size (bp)** | **No of contigs** | **G+C (mol %)** | **N50 (bp)** | **CDS (Protein)** | **Genes assigned to COGs** | **Genes assigned to KEGG** |
| --- | --- | --- | --- | --- | --- | --- | --- | --- | --- | --- |
| *Streptomyces* sp. G1^T^ | JAMOZA000000000 | Illumina HiSeq | 100× | 14173392 | 182 | 71.66 | 109952 | 12999 | 9379 | 4190 |
| *Streptomyces* sp. G2^T^ | JAMOZB000000000 | Illumina HiSeq | 100× | 8746120 | 75 | 73.34 | 204170 | 7853 | 5915 | 2914 |
| *Streptomyces* sp. G3^T^ | JAMOZC000000000 | Illumina HiSeq | 100× | 8089599 | 45 | 72.21 | 328641 | 7283 | 5584 | 2800 |
