# Supplementary figures and images for: Characterizing corn-straw-degrading actinomycetes and evaluating application efficiency in straw-returning experiments
Source: Front Microbiol. 2022 Dec 5;13:1003157. doi: 10.3389/fmicb.2022.1003157 (PMC9760696; doi:10.3389/fmicb.2022.1003157)

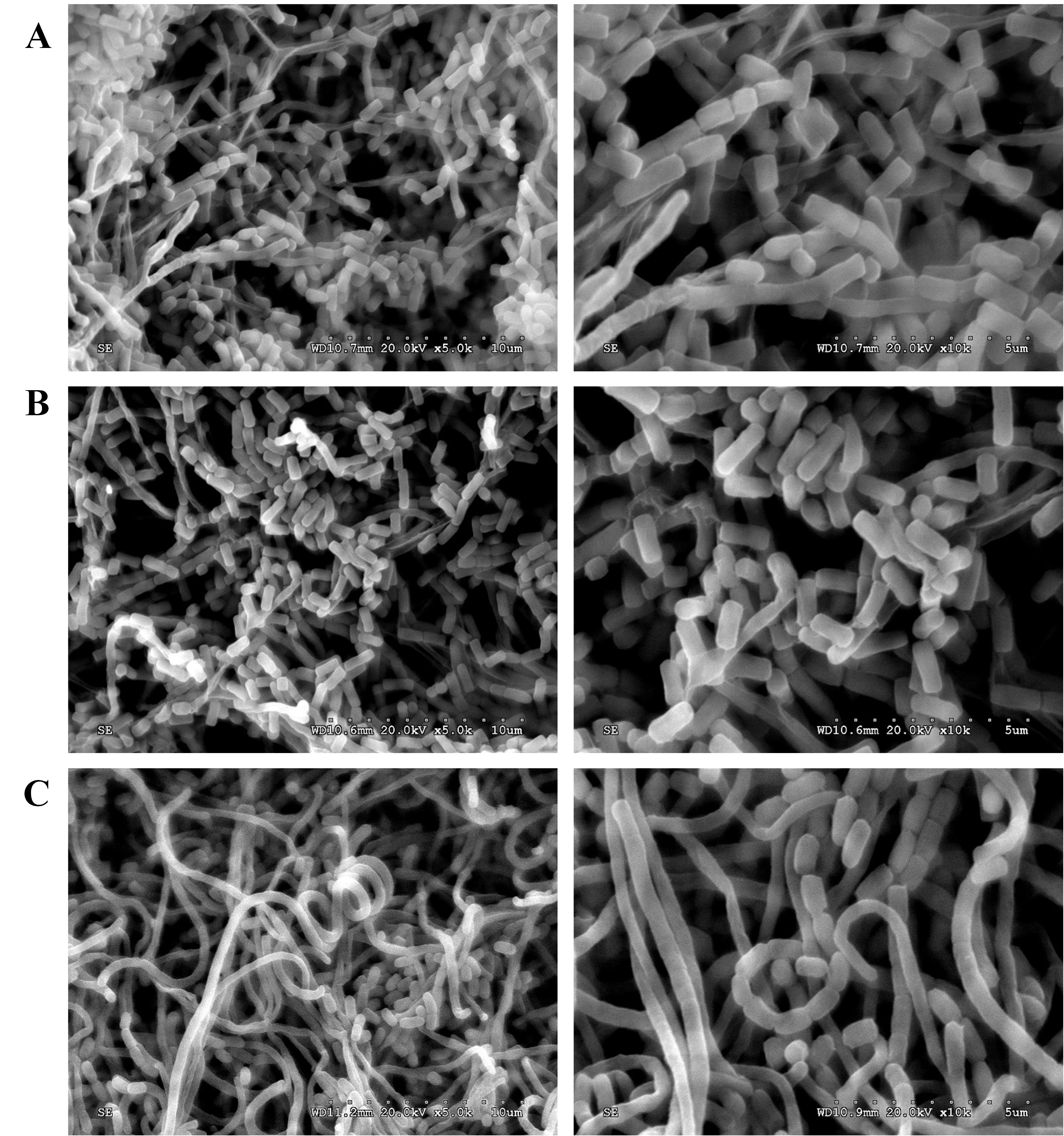

Supplement: Supplementary file 4 [file Image_1.TIF]

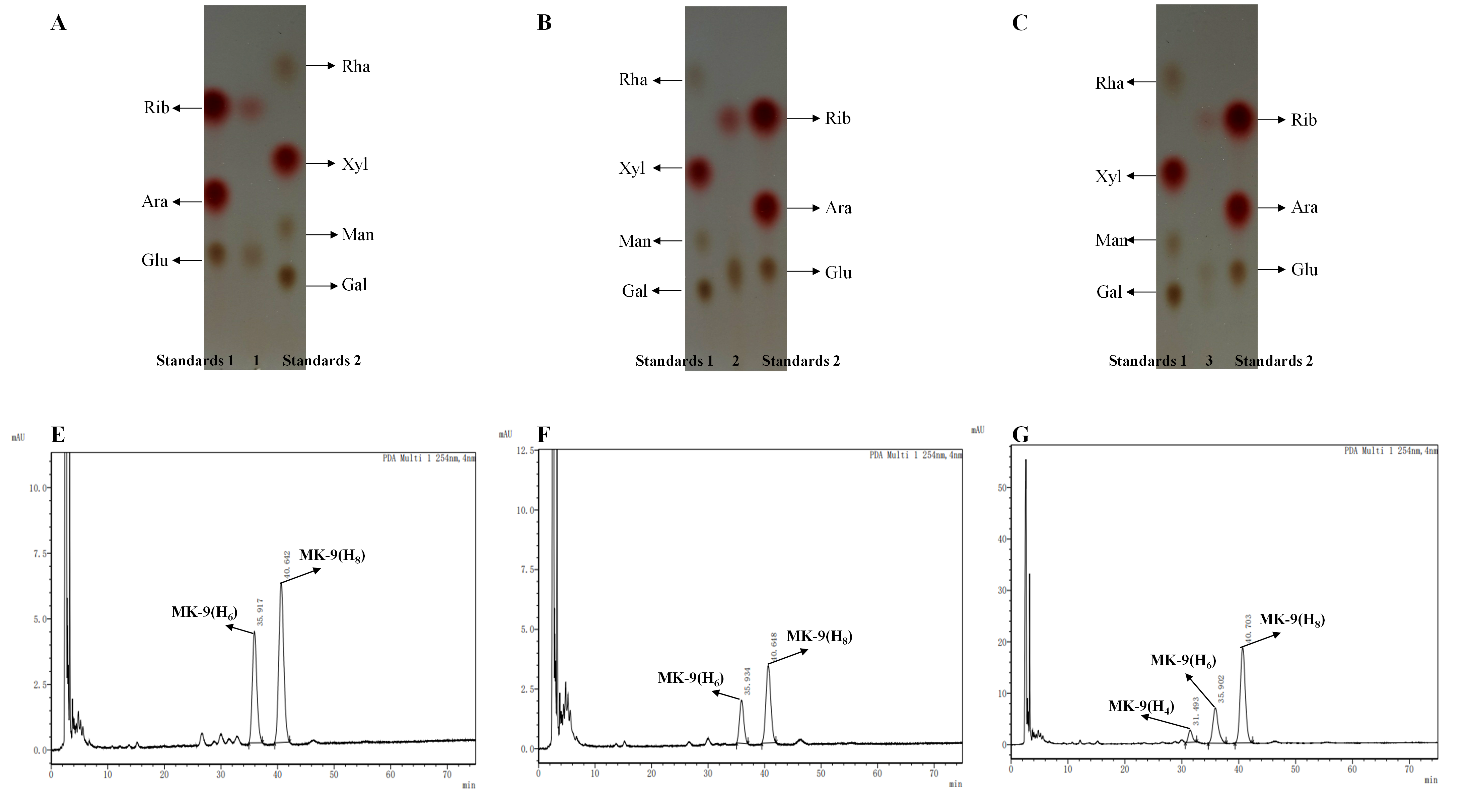

Supplement: Supplementary file 5 [file Image_2.TIF]

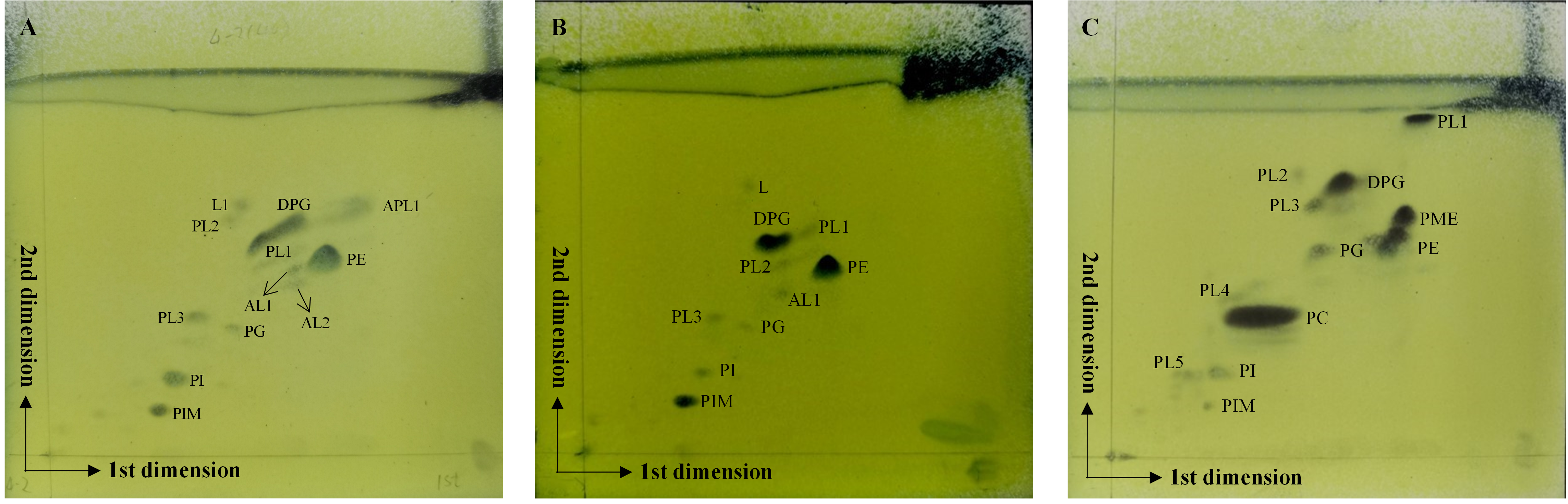

Supplement: Supplementary file 6 [file Image_3.TIF]

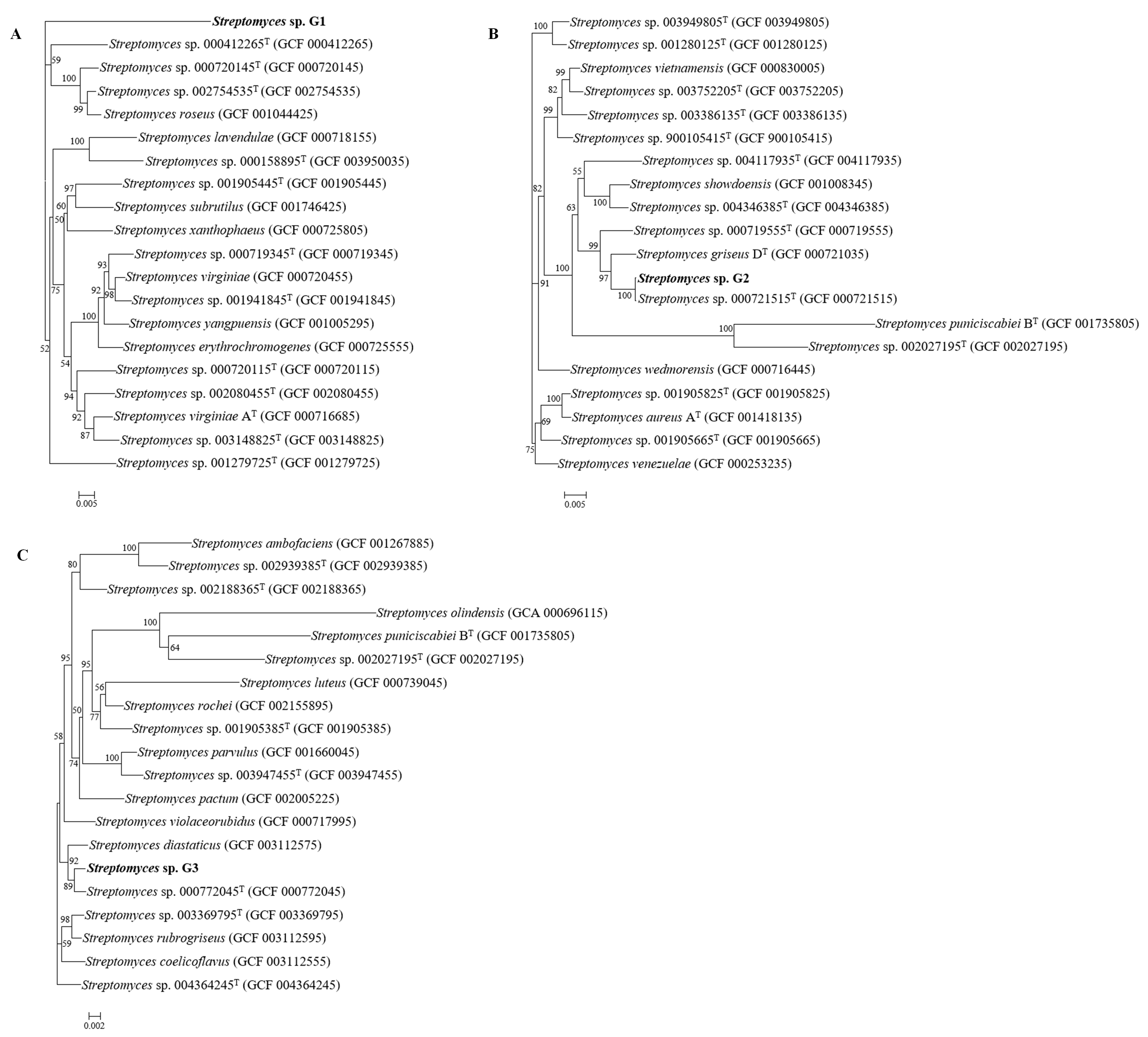

Supplement: Supplementary file 7 [file Image_4.TIF]

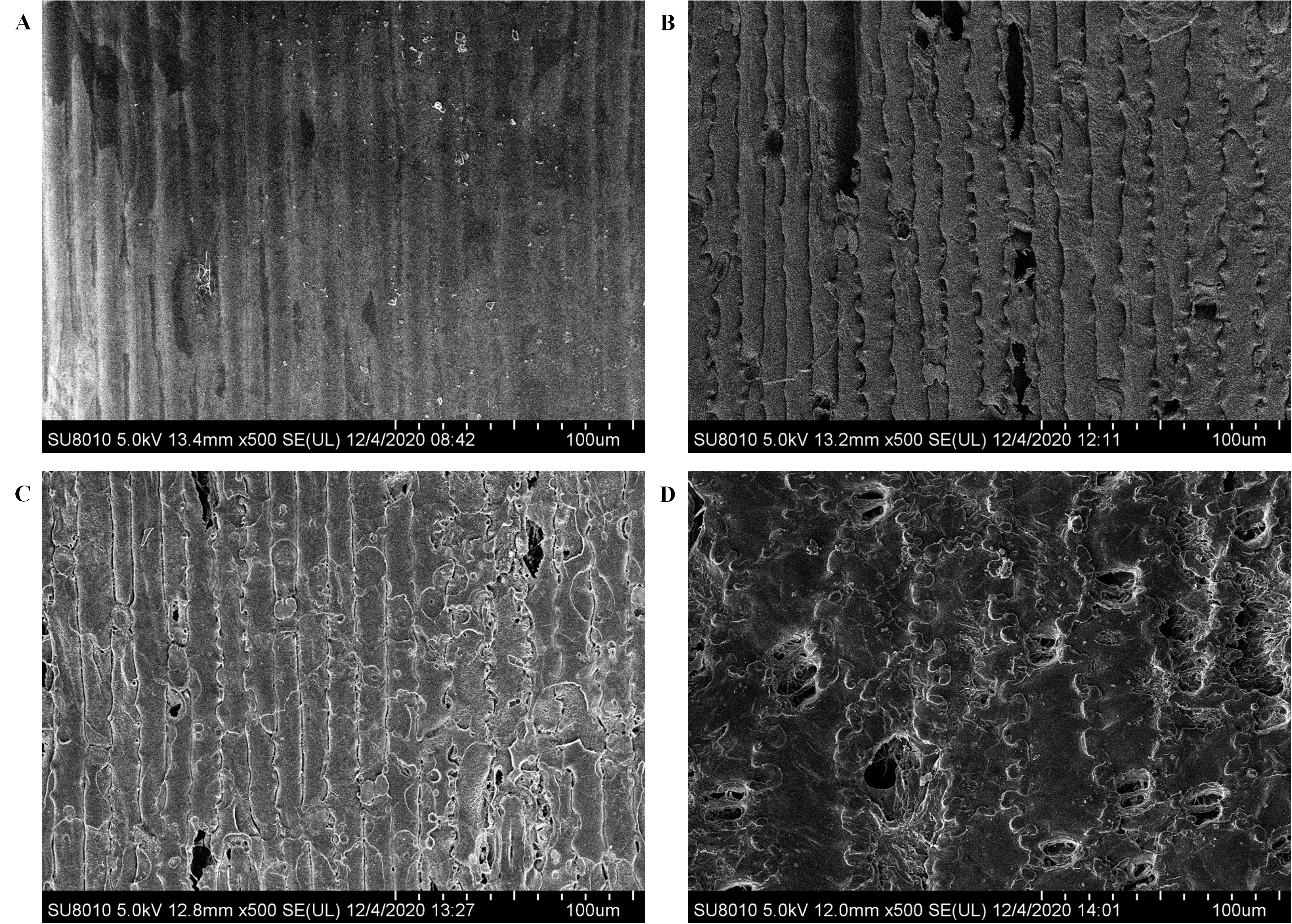

Supplement: Supplementary file 8 [file Image_5.TIF]

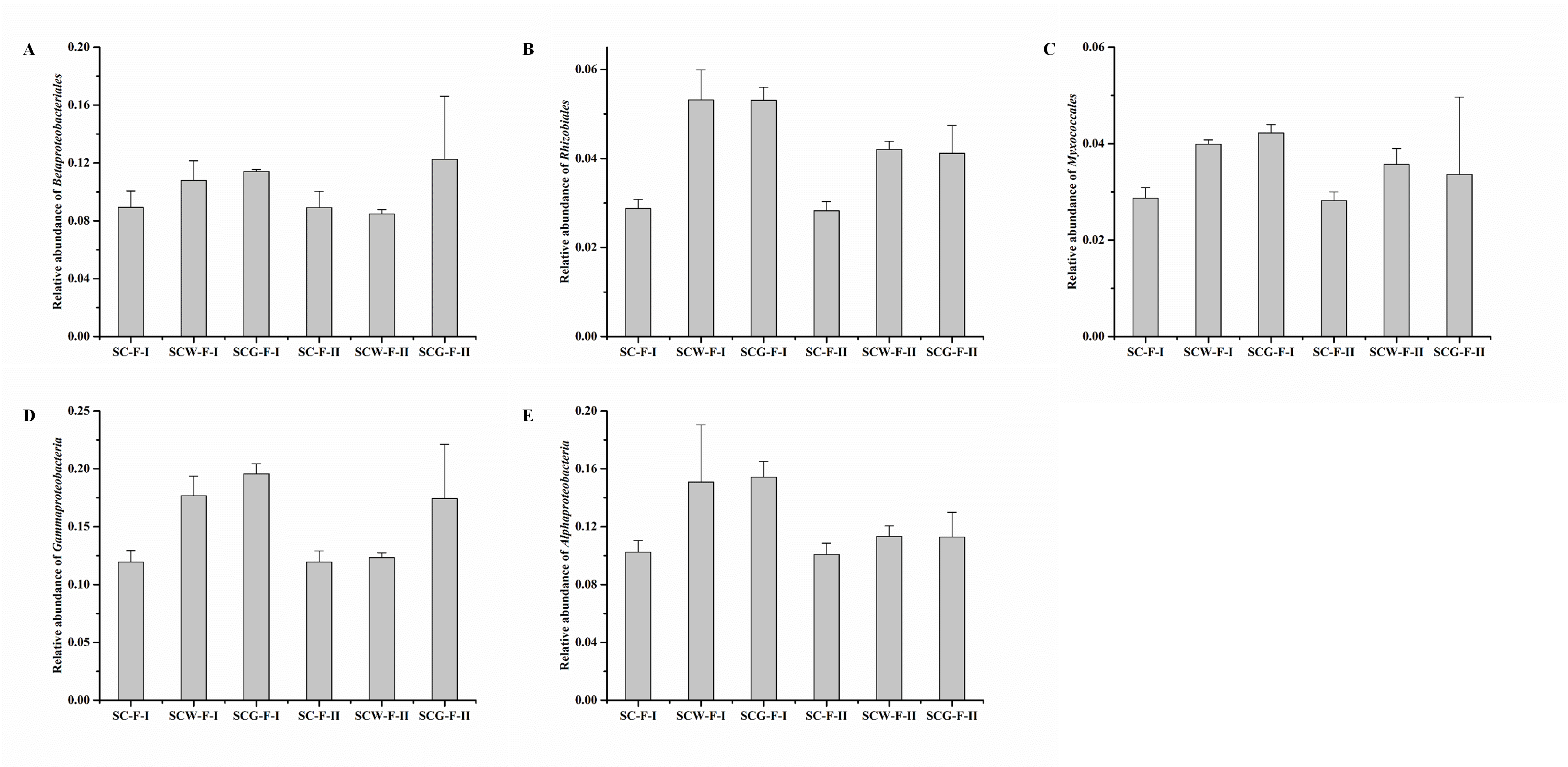

Supplement: Supplementary file 9 [file Image_6.TIF]
